# Tyrosine phosphorylation of DEPTOR functions as a molecular switch to activate mTOR signaling

**DOI:** 10.1016/j.jbc.2021.101291

**Published:** 2021-10-09

**Authors:** Laurence M. Gagné, Nadine Morin, Noémie Lavoie, Nicolas Bisson, Jean-Philippe Lambert, Frédérick A. Mallette, Marc-Étienne Huot

**Affiliations:** 1 Centre de Recherche sur le Cancer de l’Université Laval, Québec, Quebec, Canada; 2 Centre de Recherche du CHU de Québec-Université Laval, Québec, Quebec, Canada; 3 PROTEO - Regroupement québécois de recherche sur la fonction, l’ingénierie et les applications des protéines, Québec, Quebec, Canada; 4 Département de Biologie moléculaire, biochimie médicale et pathologie, Université Laval, Québec, Quebec, Canada; 5 Département de Médecine Moléculaire, Université Laval, Québec, Quebec, Canada; 6 Département de Biochimie et Médecine moléculaire, Université de Montréal, Montréal, Quebec, Canada; 7 Chromatin Structure and Cellular Senescence Research Unit, Maisonneuve-Rosemont Hospital Research Centre, Montréal, Quebec, Canada; 8 Département de Médecine, Université de Montréal, Montréal, Quebec, Canada

**Keywords:** mTOR, DEPTOR, EPHB2, tyrosine phosphorylation, β-TRCP1, β-transducin repeat–containing protein 1, CIP, calf intestinal alkaline phosphatase, EPH, erythropoietin-producing hepatocellular carcinoma, Fer, Feline sarcoma–related protein, FRQS, Fonds de Recherche du Québec—Santé, HA, hemagglutinin, IP, immunoprecipitation, mTOR, mechanistic target of rapamycin, mTORC1, mTOR complex 1, mTORC2, mTOR complex 2, PTEN, phosphatase and tensin homolog, PTM, post-translational modification, *R*-2HG, *R*-2-hydroxyglutarate, SYK, spleen tyrosine kinase, Tyr, tyrosine

## Abstract

Metabolic dysfunction is a major driver of tumorigenesis. The serine/threonine kinase mechanistic target of rapamycin (mTOR) constitutes a key central regulator of metabolic pathways promoting cancer cell proliferation and survival. mTOR activity is regulated by metabolic sensors as well as by numerous factors comprising the phosphatase and tensin homolog/PI3K/AKT canonical pathway, which are often mutated in cancer. However, some cancers displaying constitutively active mTOR do not carry alterations within this canonical pathway, suggesting alternative modes of mTOR regulation. Since DEPTOR, an endogenous inhibitor of mTOR, was previously found to modulate both mTOR complexes 1 and 2, we investigated the different post-translational modification that could affect its inhibitory function. We found that tyrosine (Tyr) 289 phosphorylation of DEPTOR impairs its interaction with mTOR, leading to increased mTOR activation. Using proximity biotinylation assays, we identified SYK (spleen tyrosine kinase) as a kinase involved in DEPTOR Tyr 289 phosphorylation in an ephrin (erythropoietin-producing hepatocellular carcinoma) receptor–dependent manner. Altogether, our work reveals that phosphorylation of Tyr 289 of DEPTOR represents a novel molecular switch involved in the regulation of both mTOR complex 1 and mTOR complex 2.

The serine/threonine kinase mechanistic target of rapamycin (mTOR) is a central regulator of cellular metabolism, and its dysfunction is frequently observed in cancer. Indeed, constitutive activation of mTOR-regulated pathways is now considered as a potent inducer of tumor growth and cancer cell survival (1, 2, 3, 4, 5, 6). mTOR constitutes the core component of two protein complexes, mTOR complex 1 (mTORC1) and mTOR complex 2 (mTORC2). mTOR, mLST8, and DEPTOR are common to each complex, whereas some components are specific to mTORC1 or mTORC2. RAPTOR and PRAS40 are also forming mTORC1, whereas RICTOR, mSIN1, and PROTOR are assembled into mTORC2 (7, 8, 9, 10, 11, 12). These exclusive components confer each complex with specific activities, such as cell proliferation and autophagy for mTORC1, and cell survival and cytoskeletal rearrangement for mTORC2 (13).

mTOR functions as a central metabolic regulator involved in the modulation of cellular processes, such as autophagy, macromolecule biosynthesis, lysosome biogenesis, cell mobility, cell survival, and cytoskeletal rearrangement (14). The involvement of mTOR in these diverse cellular processes is regulated by a vast array of cellular contexts, including hypoxia, inflammation, energetic variation, growth factor stimulation, and nutrient availability. Because of its biological versatility, it is not surprising to observe altered mTOR activation in many cellular dysfunctions and diseases (13). Thus, mTOR has to be tightly regulated to maintain cellular homeostasis, a function performed by the DEP domain–containing mTOR-interacting protein (DEPTOR), an endogenous mTORC1 and mTORC2 inhibitor (12).

As aforementioned, mTOR is frequently activated in human cancers following mutation in the phosphatase and tensin homolog (PTEN)/PI3K/AKT canonical activation pathway (15, 16). However, some cancers are not carrying mutations within this pathway but still harbor constitutive activation of mTOR (17, 18). This is the case with approximately 85% of low-grade glioma that are associated with isocitrate dehydrogenase 1 or 2 mutations (19). These mutations allow the production of *R*-2-hydroxyglutarate (*R*-2HG), an oncometabolite, instead of their normal product, α-ketoglutarate (20, 21). Because of their molecular similarity, *R*-2HG can inhibit the catalytic activities of numerous α-ketoglutarate-dependent enzymes (22), resulting in the massive degradation of DEPTOR, the endogenous repressor of mTOR (23). *R*-2HG-induced DEPTOR degradation leads to constitutive mTORC1 and mTORC2 activation in a PTEN/PI3K/AKT-independent manner (23). Moreover, many post-translational modifications (PTMs) were identified to affect DEPTOR inhibitory activity on mTOR. For example, DEPTOR was shown to be serine phosphorylated (Ser^286/287/291/293/299^) by mTOR and CK1α under growth factor stimulation, leading to its degradation by the ubiquitin/proteasome system (24, 25, 26). While it is clear that DEPTOR degradation can affect mTOR-associated biological processes and pathology, the precise mechanisms underlying DEPTOR endogenous modulation allowing the fine-tuning of mTOR activity remained unexplored.

Erythropoietin-producing hepatocellular carcinoma (EPH) receptors are the largest family of receptor protein tyrosine kinase. They are involved in a vast variety of cellular processes, such as proliferation, differentiation, cell shape, and mobility (27). Their dysregulation is often associated with cancers and developmental disorders (28). The EPH receptors are divided in either type A subgroup that contains receptors EPHA1–A8 and EPHA10, or the type B subgroup, comprising receptors EPHB1–B4 and EPHB6. Aside from the pseudokinases EPHA10 and EPHB6, all EPH receptors display catalytic activity (29, 30). Their ligands are ephrins, which are also divided into subclasses A and B, based on their membrane attachment. Ephrins A are anchored by a glycosylphosphatidylinositol linkage, whereas ephrins B have a transmembrane domain and a C-terminal PDZ domain (31). While some degree of crosstalk is documented, EPHA receptors bind preferentially to ephrins A and EPHB receptors to ephrins B (32). EPH receptor activation is mainly triggered by cell–cell contact or by an extracellular signaling from neighboring cells. The complexity of EPH receptor–ephrin signaling results in a vast variety of functions and regulation. For this reason, dysregulation of the expression of EPH receptors is often associated with cancer (33, 34).

We identified a novel phosphorylation event targeting DEPTOR tyrosine (Tyr) 289, which rapidly affects its inhibitory function on both mTORC1 and mTORC2. Phosphorylation of DEPTOR on Tyr 289 weakens its association with mTOR, thus allowing a rapid and sustained activation of both mTORC1 and mTORC2. Proximity biotinylation experiments identified SYK (spleen tyrosine kinase) as a kinase implicated on DEPTOR Tyr phosphorylation. Using combinatory methods, we found that the EPHB2 receptor signaling pathway also promotes DEPTOR Tyr 289 phosphorylation through SYK activation.

Taken together, our findings identified a novel molecular mechanism involved in modulating mTOR activity and a previously unidentified potential pathway contributing to PTEN/PI3K/AKT-independent increase of mTOR activity observed in a subset of cancers.

## Results

### The mTOR inhibitor DEPTOR is Tyr phosphorylated

**Figure 1:**
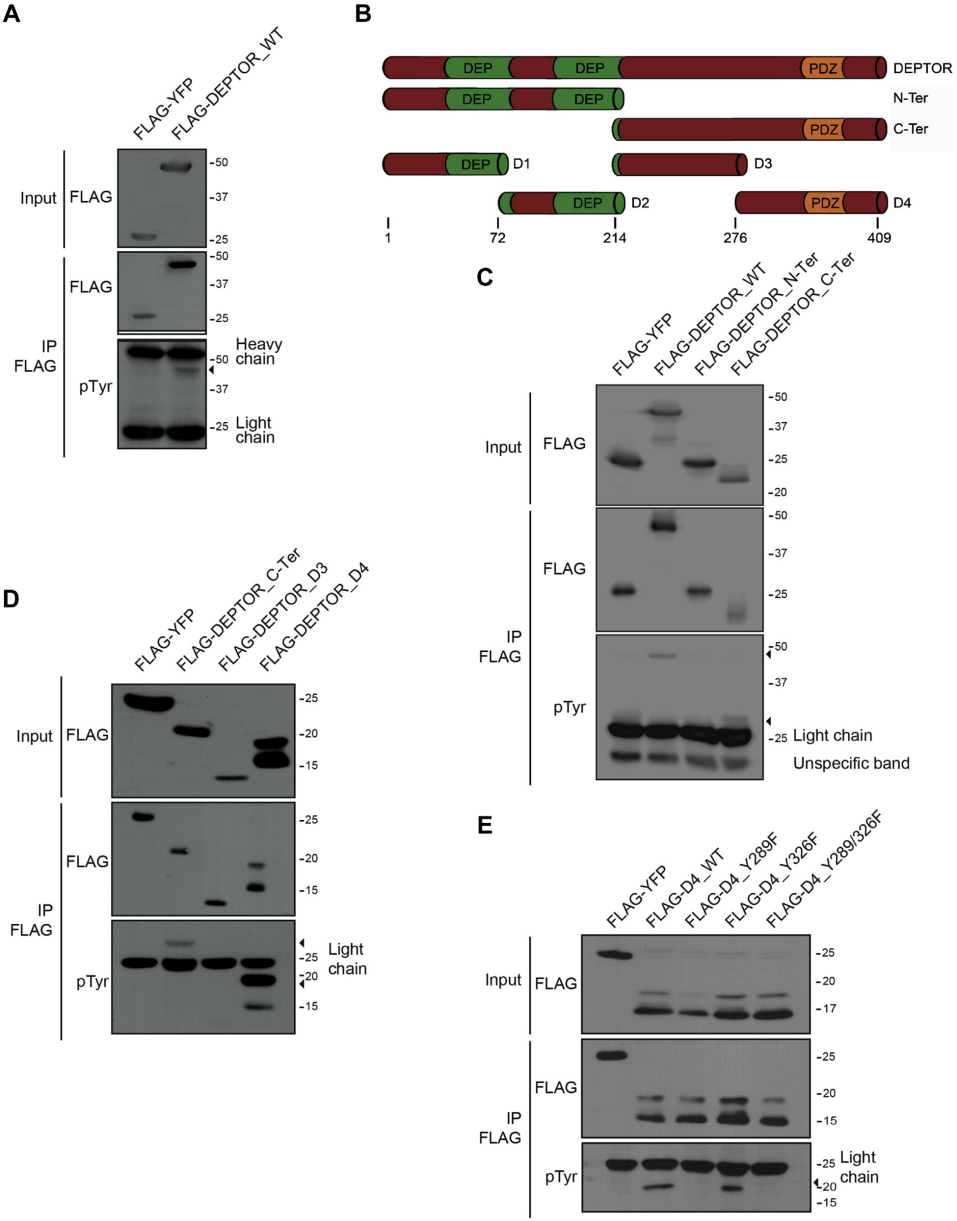
**DEPTOR is phosphorylated on tyrosine 289.***A*, denaturing FLAG immunoprecipitation of 293T cells transfected with FLAG-YFP (negative control) or FLAG-DEPTOR. DEPTOR tyrosine phosphorylation was detected using antiphosphotyrosine antibodies (4G10). *B*, schematic representation of the different DEPTOR fragments. *C*, 293T cells transfected with FLAG-YFP (negative control), FLAG-DEPTOR^WT^, FLAG-DEPTOR N-terminal region, or FLAG-DEPTOR C-terminal region were lysed for immunoprecipitation in denaturing condition. Immunoprecipitated DEPTOR phosphorylation was assessed using antiphosphotyrosine antibody 4G10. *D*, 293T cells transfected by FLAG-YFP (negative control), FLAG-DEPTOR C-terminal region, FLAG-DEPTOR D3, and FLAG-DEPTOR D4 were lysed for immunoprecipitation in denaturing condition. Immunoprecipitated DEPTOR phosphorylation was assessed using antiphosphotyrosine antibody 4G10. *E*, DEPTOR tyrosine phosphorylation is located on the residue 289. 293T cells were transfected by FLAG-YFP (negative control), FLAG-DEPTOR-D4^WT^, FLAG-DEPTOR-D4^Y289F^, FLAG-DEPTOR-D4^Y326F^, or FLAG-DEPTOR-D4^Y289-326F^, and a denaturing immunoprecipitation was made to evaluate their phosphorylation status.

Increasing evidence is pointing toward multiple PTMs playing a disruptive role on DEPTOR function as an endogenous inhibitor of mTOR. While serine phosphorylation was shown to trigger DEPTOR proteasomal degradation (12, 24, 25, 26), no PTM has been identified as a reversible molecular switch to modulate DEPTOR functions and in turn rapidly activate/inhibit mTORC1 and mTORC2. We hypothesized that DEPTOR is Tyr phosphorylated following growth factor stimulation and that this regulates DEPTOR stability and/or activity. We first used a phospho-Tyr-specific antibody (4G10) on denatured DEPTOR immunoprecipitate extract and were able to detect a Tyr phosphorylation event on DEPTOR (Fig. 1*A*).

Since Tyr phosphorylation is known to modulate protein interactions and activity in a rapid and reversible manner (35), we sought to map DEPTOR residue(s) undergoing Tyr phosphorylation. To do this, we assessed if Tyr phosphorylation was restricted to a specific portion of DEPTOR. Using different fragments of DEPTOR (Fig. 1*B*), we found Tyr phosphorylation on the C-terminal half of the protein (amino acids 214–409) (Fig. 1*C*). We further divided the C-terminal fragment in two smaller fragments (D3 and D4 fragments) and found that Tyr phosphorylation was restricted to the D4 fragment (amino acids 276–409) (Fig. 1*D*).

We observed multiple bands above the major band for DEPTOR C-terminal and DEPTOR D4 fragment (Fig. 1, *C* and *D*). For both of them, the upper band seems to be the one harboring most of the Tyr phosphorylation. We then investigated the nature of this bands pattern by using a DEPTOR D4 fragment tagged on both its N terminus (FLAG) and C terminus (hemagglutinin [HA]). Detection of both bands of DEPTOR fragment D4 using FLAG and HA antibodies suggested that these forms of DEPTOR are not resulting from proteolytic cleavage but rather resulting of PTMs (Fig. S1*A*). Only the higher molecular weight band of DEPTOR fragment D4 was Tyr phosphorylated and recognized using 4G10 antibody (Fig. S1*B*). In addition, we validated that the ∼18 kDa band was not solely the result of DEPTOR Tyr phosphorylation, since it was still observable using anti-FLAG in Tyr phosphatase-treated immunoprecipitates (Fig. S1*B*). Sequence alignment revealed that three tyrosines are present within D4 fragment and that only two are evolutionarily conserved, namely Tyr 289 and Tyr 326 (Fig. S1*C*). By generating point mutation of each conserved tyrosine within the D4 fragment into nonphosphorylatable phenylalanine (Y to F), we determined that only Tyr 289 can be phosphorylated (Fig. 1*E*).

### DEPTOR Tyr phosphorylation modulates its association with mTOR

**Figure 2:**
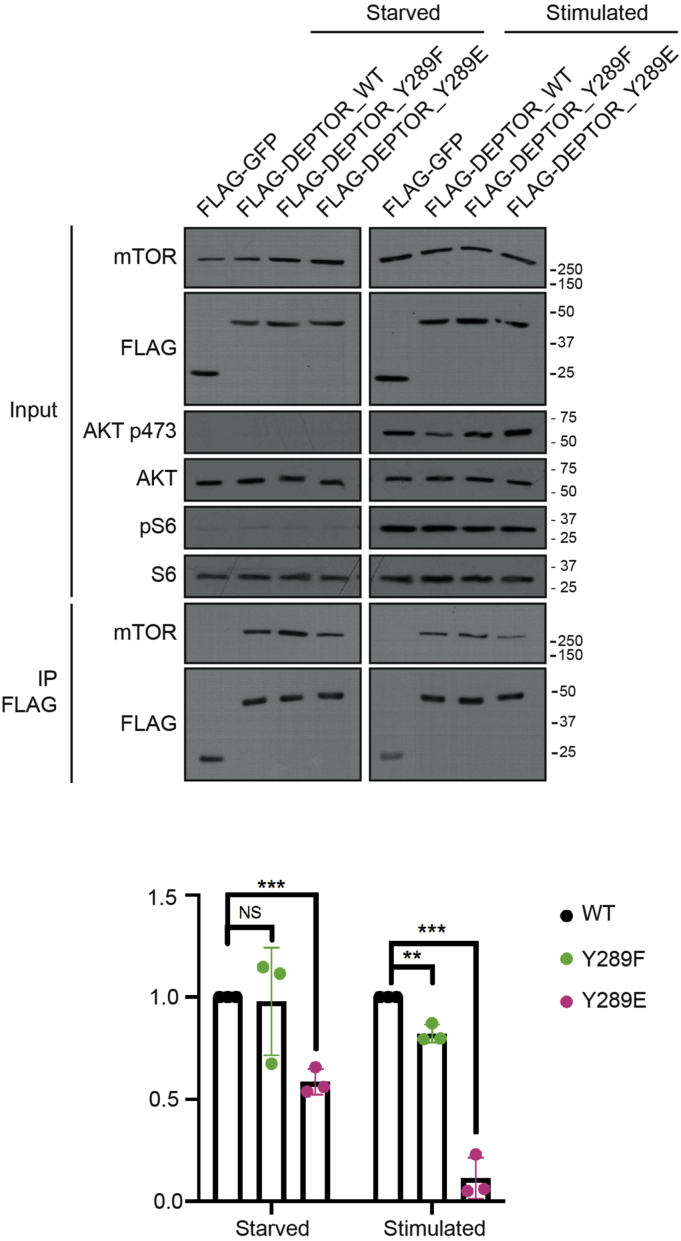
**DEPTOR tyrosine phosphorylation reduces the affinity of mTOR complexes.** DEPTOR-depleted HeLa cells were infected with FLAG-GFP, FLAG-DEPTOR^WT^, FLAG-DEPTOR^Y289F^, or FLAG-DEPTOR^Y289E^. Lysates from serum-starved cells and serum-starved cells followed by serum stimulation with complete media (2 h) were used for immunoprecipitation against FLAG-tagged proteins, and associated proteins were revealed by Western blots. mTOR, mechanistic target of rapamycin.

DEPTOR association to mTOR is mediated by their respective PDZ (DEPTOR) and FAT (mTOR) domain (12). We investigated whether phosphorylation on Tyr 289, which is within the PDZ domain, could be involved in modulating DEPTOR ability to bind mTOR. The dynamics of the DEPTOR–mTOR association are regulated through energy levels within the cell, as a low energy level favors tight binding between DEPTOR and mTOR, while high nutrient availability promotes their dissociation (24, 25, 26). To assess the impact of Tyr 289 phosphorylation on DEPTOR binding to mTOR, we starved cells prior to serum stimulation and then performed DEPTOR immunoprecipitation (IP). Endogenous DEPTOR was first depleted from HeLa cells using validated DEPTOR shRNAs targeting the 3′UTR of DEPTOR (Fig. S2*A*). We then infected these cells with shRNA-resistant WT DEPTOR or mutants harboring Tyr 289 phosphomimetic Y to E substitution (DEPTOR^Y289E^) or a nonphosphorylatable Y to F substitution (DEPTOR^Y289F^). In starved cells, nonphosphorylatable DEPTOR^Y289F^ showed increased association to mTOR when compared with DEPTOR^WT^, whereas the phosphomimetic version of DEPTOR (DEPTOR^Y289E^) showed a slight decreased binding when compared with DEPTOR^WT^ (Fig. 2). This difference in association of DEPTOR^Y289E^ was even more noticeable following growth factor stimulation, while both DEPTOR^WT^ and DEPTOR^Y289F^ showed similar association to mTOR (Fig. 2). These results suggest that Tyr 289 phosphorylation negatively modulates DEPTOR ability to bind mTOR in serum-starved and serum-stimulated conditions. Thus, by modulating DEPTOR–mTOR association, DEPTOR phosphorylation on Y289 might then promote mTOR activation.

### DEPTOR Tyr 289 phosphorylation modulates mTOR activity

**Figure 3:**
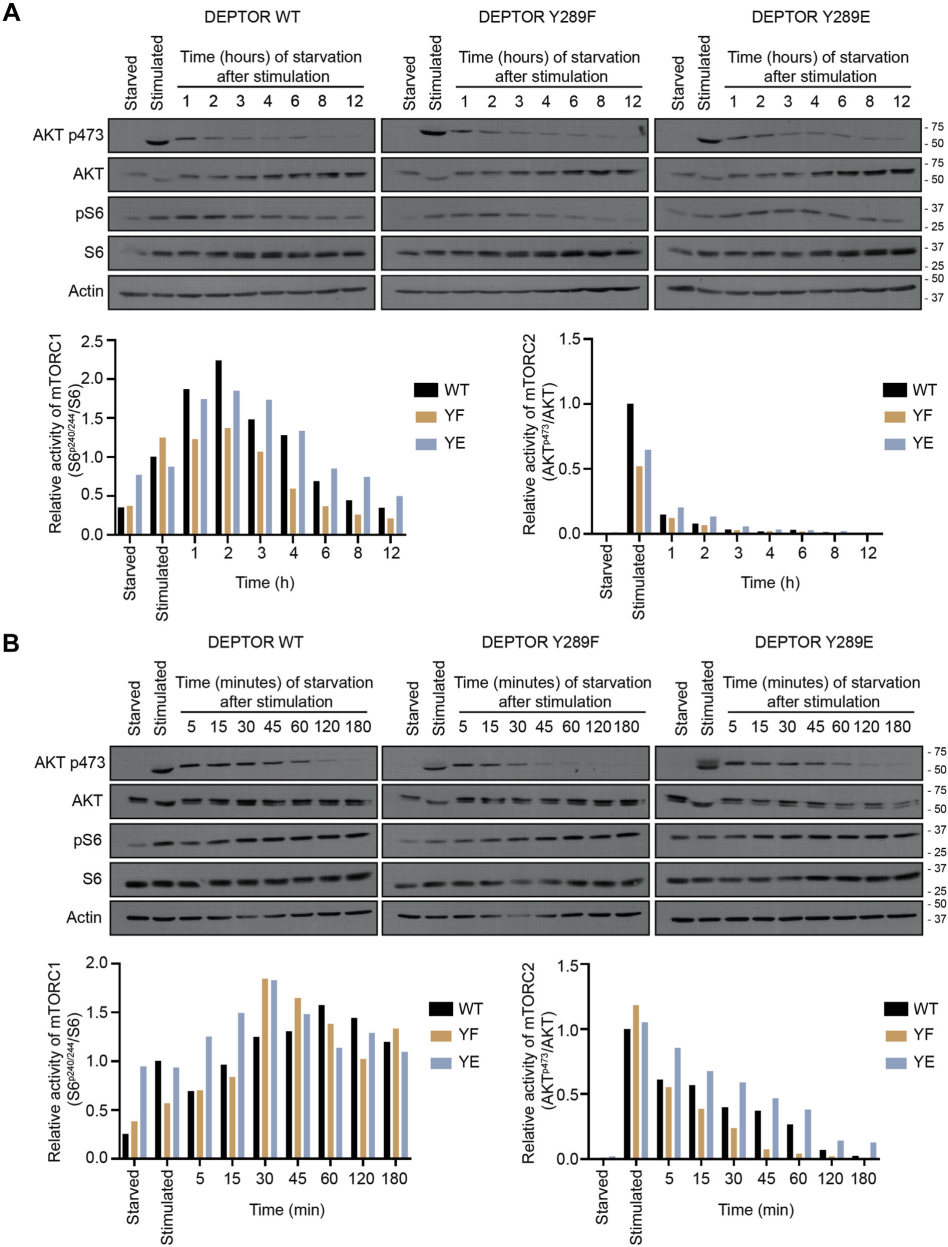
**DEPTOR tyrosine 289 phosphorylation impairs its inhibitory functions.***A*, DEPTOR-depleted HeLa cells were infected with FLAG-DEPTOR^WT^, FLAG-DEPTOR^Y289F^, or FLAG-DEPTOR^Y289E^. Cells were starved 16 h, then stimulated with complete media for 30 min, and starved again for 1, 2, 3, 4, 6, 8, or 12 h. mTORC1/mTORC2 activation was assessed using Western blot for each time points (*upper panels*) and quantified (*lower panels*). *B*, DEPTOR-depleted HeLa cells were infected with FLAG-DEPTOR^WT^, FLAG-DEPTOR^Y289F^, or FLAG-DEPTOR^Y289E^. Cells were starved 16 h before being stimulated with complete media for 30 min and starved again for 5, 15, 30, 45, 60, 120, or 180 min. The lysates were blotted to see mTORC1/mTORC2 activation using Western blot for each time point (*upper panels*) and quantified (*lower panels*). mTORC1, mechanistic target of rapamycin complex 1; mTORC2, mechanistic target of rapamycin complex 2.

Growth factor–induced DEPTOR dissociation from mTOR was shown to positively modulate mTOR activity (24, 25, 26). To determine whether DEPTOR Tyr phosphorylation impacts mTOR activity, we investigated the repressive function of DEPTOR phosphorylation mutant on mTORC1 (phospho-S6^S240/244^) and mTORC2 (phospho-AKT^S473^) activation. To monitor the regulation of mTOR activity, cells were first serum starved for 16 h, then stimulated with complete media for 30 min, and then replaced with starving media for 12 h in order to follow kinetics of the regulation of mTOR activity. We observed a faster decrease in phosphorylation of S6 in DEPTOR^Y289F^-expressing cells, compared with cells expressing DEPTOR^WT^, whereas those expressing DEPTOR^Y289E^ showed a sustained mTORC1 activation until 4 h poststimulation (Fig. 3*A*), suggesting that phosphorylation on Tyr 289 impairs DEPTOR ability to suppress mTORC1 activity. All DEPTOR mutants showed similar behavior regarding phospho-AKT^S473^, a readout for mTORC2 activation, after serum stimulation, followed by rapid decrease of phosphorylated AKT within the first hour (Fig. 3*A*). Because of the faster regulation of mTORC2 following stimulation, phospho-AKT^S473^ levels in mutant DEPTOR-expressing cells was rather determined within the first hour after serum stimulation. Like mTORC1 activity, cells expressing DEPTOR^Y289F^ mutant showed a faster decrease of phospho-AKT^S473^ compared with cells expressing DEPTOR^WT^, whereas DEPTOR^Y289E^-expressing cells displayed sustained mTORC2 activation following serum removal (Fig. 3*B*). These results confirmed that DEPTOR phosphorylation on Tyr 289 decreases its inhibitory functions on mTOR activity.

### DEPTOR Tyr 289 phosphorylation affects mTORC2-dependent cellular functions

**Figure 4:**
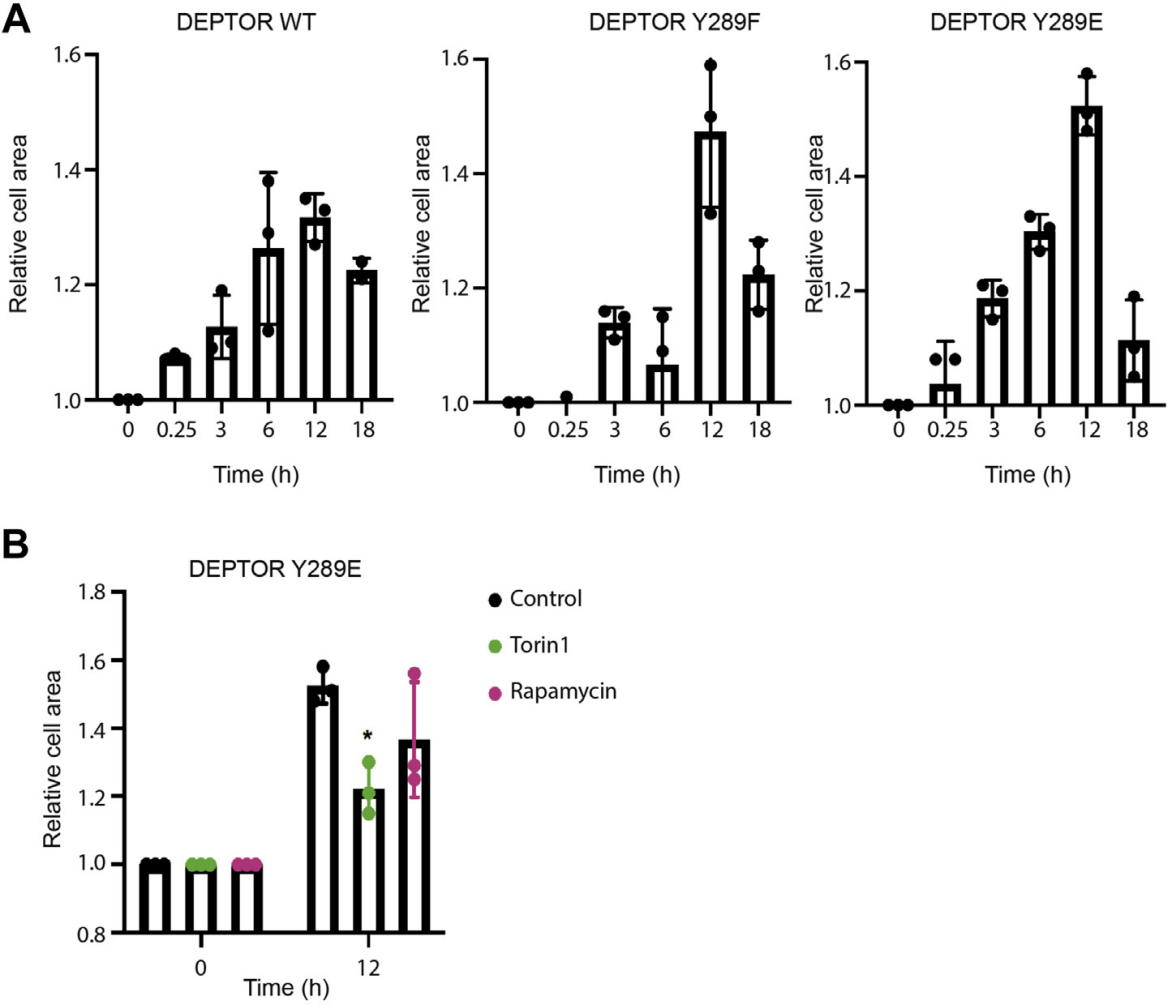
**DEPTOR tyrosine phosphorylation increases mTOR activity.***A*, DEPTOR-depleted HeLa cells were infected with FLAG-DEPTOR^WT^, FLAG-DEPTOR^Y289F^, and FLAG-DEPTOR^Y289E^ and starved for 16 h. Cells were then stimulated for 15 min, 3, 6, 12, and 18 h. Mean cell area was measured using NIS-Elements for each condition (≈200 cells per condition). *B*, DEPTOR-depleted HeLa cells were infected with FLAG-DEPTOR^Y289E^, starved for 16 h, and stimulated for 12 h with rapamycin (10 μM) or Torin 1 (1 μM). Mean cell area was measured using NIS-Elements for each condition (≈200 cells per condition). mTOR, mechanistic target of rapamycin.

To further investigate the cellular impact of DEPTOR Tyr 289 phosphorylation on mTOR-dependent mechanisms, we assessed its effect on cytoskeletal reorganization by measuring cell size area upon stimulation. We measured cell size area of HeLa cells depleted of endogenous DEPTOR expressing DEPTOR^WT^, DEPTOR^Y289F^, or DEPTOR^Y289E^ at different time points for 18 h after growth factor stimulation (Fig. 4*A*). Cell area rapidly increased 1 to 3 h following stimulation and reached maximum size at 12 h poststimulation. The increase in cell area was even more noticeable in cells expressing DEPTOR^Y289E^ comparatively to cells expressing either DEPTOR^WT^ or nonphosphorylatable DEPTOR^Y289F^ (Fig. 4*A*). These results are consistent with our assessment of mTORC1 and mTORC2 activation (Fig. 3). Moreover, DEPTOR^Y289F^ was found to be more potent than DEPTOR^WT^ to repress mTOR activity, suggesting that the phosphorylation status of Tyr 289 could directly affect the ability of DEPTOR to bind and repress activated mTOR. To confirm the specific involvement of mTORC2 in this process, we compared the effect of rapamycin (inhibitor of mTORC1) and Torin 1 (inhibitor of both mTOR complexes) on growth factor–induced cytoskeletal modulation of cells expressing DEPTOR^Y289E^. We observed a significant decrease in cell area upon treatment with Torin 1. When only mTORC1 was inhibited, the decrease in cell area was less marked, confirming the impact of mTORC2 on cell area (Fig. 4*B*). These results confirm that DEPTOR phosphorylation on Tyr 289 sustained mTORC2 activity leading to increased cytoskeletal reorganization.

### Tyr 289 phosphorylation increases DEPTOR stability

**Figure 5:**
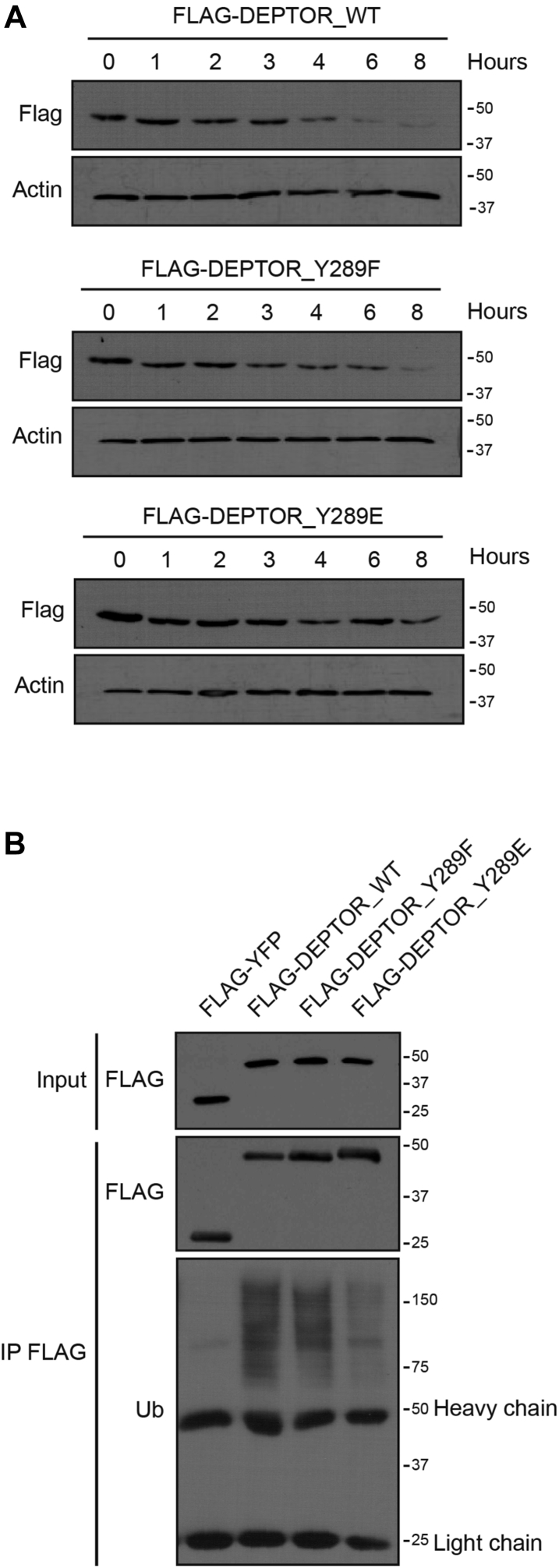
**DEPTOR Tyr**^**289**^**phosphorylation increases its stability.***A*, DEPTOR-depleted HeLa cells were infected with FLAG-DEPTOR^WT^, FLAG-DEPTOR^Y289F^, or FLAG-DEPTOR^Y289E^, were starved for 16 h, pretreated with 50 μg/ml of cycloheximide for 1 h, and stimulated with complete media supplemented with cycloheximide (50 μg/ml) for 0, 1, 2, 3, 4, 6, and 8 h. *B*, 293T cells transfected with FLAG-YFP, FLAG-DEPTOR^WT^, FLAG-DEPTOR^Y289F^, or FLAG-DEPTOR^Y289E^ were stimulated 30 min with insulin (100 nM) and lysed for immunoprecipitation in denaturing condition. Ubiquitination was assessed by Western blot.

Ubiquitin-mediated degradation of DEPTOR is an important molecular process allowing the release of mTOR from DEPTOR-mediated inhibition (24, 25, 26). Thus, we assessed to determine whether Tyr 289 phosphorylation affected DEPTOR protein stability by performing cycloheximide chase experiments following serum stimulation. We found that DEPTOR^Y289E^ was more stable through time than DEPTOR^WT^ and the unphosphorylated version DEPTOR^Y289F^ (Fig. 5*A*). To assess if DEPTOR Tyr 289 phosphorylation was preventing its degradation, we immunoprecipitated each construct and measured their respective ubiquitination following insulin stimulation. We observed similar ubiquitination patterns for DEPTOR^WT^ and DEPTOR^Y289F^, whereas ubiquitination of DEPTOR^Y289E^ was significantly decreased (Fig. 5*B*). To further confirm the effect of Tyr 289 phosphorylation on DEPTOR ubiquitination, we assessed its effect on its ability to associate with β-transducin repeat–containing protein 1 (β-TRCP1), an E3 ubiquitin ligase previously identified to be involved in DEPTOR degradation (24, 25, 26). As expected, we found that only DEPTOR^WT^ was associated to β-TRCP1, whereas DEPTOR^Y289E^ was unable to coimmunoprecipitate β-TRCP1 (Fig. S2*B*). Taken together, these results suggest that while Tyr 289 impairs the repressive function of DEPTOR, it also prevents it from β-TRCP1-induced ubiquitin-mediated degradation. Hence, this also suggests that this phosphorylation event could act as a molecular switch allowing a rapid activation/inactivation of DEPTOR repressive function on mTOR.

### Tyr inhibitors decrease DEPTOR phosphorylation

**Figure 6:**
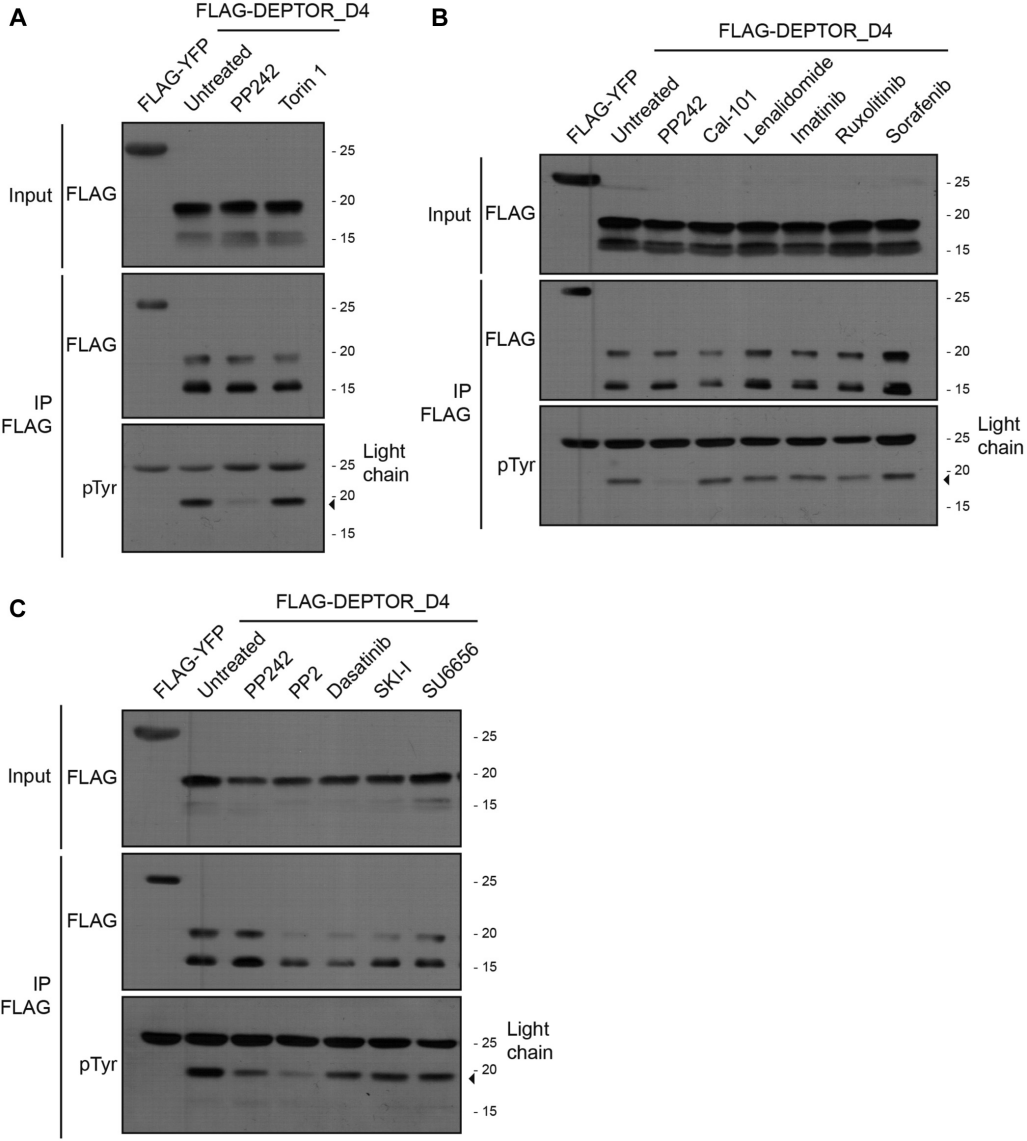
**Effect of different inhibitors on DEPTOR phosphorylation.***A*, 293T cells transfected with FLAG-YFP or FLAG-DEPTOR-D4 were treated with DMSO (untreated), PP242 (10 μM), or Torin 1 (1 μM) for 2 h. Cell lysates were used for a denaturing FLAG immunoprecipitation to reveal levels of DEPTOR phosphorylation. *B*, 293T cells transfected with FLAG-YFP or FLAG-DEPTOR-D4 were treated with DMSO (untreated), PP242 (10 μM), Cal-101 (1 μM), lenalidomide (1 μM), imatinib (10 μM), ruxolitinib (10 μM), or sorafenib (10 μM) for 2 h. Cell lysates were used for a denaturing FLAG immunoprecipitation to reveal levels of DEPTOR phosphorylation. *C*, 293T cells transfected with FLAG-YFP or FLAG-DEPTOR D4 were treated with DMSO (untreated), PP242 (10 μM), PP2 (10 μM), dasatinib (10 μM), SKI-I (10 μM), or SU6656 (10 μM) for 2 h. Cell lysates were used to perform a denaturing FLAG immunoprecipitation to reveal levels of DEPTOR phosphorylation. DMSO, dimethyl sulfoxide; SKI-I, SRC kinase inhibitor I.

We demonstrated that DEPTOR Tyr 289 phosphorylation allows a rapid relief of DEPTOR-mediated inhibition of mTOR (Fig. 3, *A* and *B*). DEPTOR Tyr 289 lies closely to serine residues phosphorylated by mTOR (Ser 293 and 299) (24, 25, 26). We then determined whether mTOR activity impacts DEPTOR Tyr 289 phosphorylation. Unexpectedly, inhibitor of both mTOR complexes, PP242 or Torin 1, showed distinct effects on DEPTOR D4 fragment Tyr 289 phosphorylation (Fig. 6*A*). While Torin 1 showed no effect, PP242 provoked a robust reduction in DEPTOR Tyr 289 phosphorylation. As PP242 is known to lose specificity at higher concentration, we tested a range of PP242 concentrations and observed inhibition of DEPTOR Tyr phosphorylation only at higher concentrations of 5 to 10 μM (Fig. S3*A*). These results suggested that the observed effect was not caused by mTOR inhibition but by another kinase inhibited at higher PP242 concentrations.

To narrow down the potential candidate kinases responsible for DEPTOR Tyr 289 phosphorylation, we tested other pharmacological inhibitors sharing some specificity toward PP242 secondary targets (Table S1) (36, 37). We first treated cells with Cal-101 and lenalidomide, inhibitors of subunits of PI3K and CK1α, respectively (Fig. 6*B*). While both kinases are known to participate in DEPTOR regulation, PI3K through the canonical pathway of mTOR (13) and CK1α *via* proteasome-mediated degradation of DEPTOR (24, 26), neither had an effect on DEPTOR Tyr phosphorylation. Treatment with imatinib (Abl and platelet-derived growth factor receptor), ruxolitinib (Jak2), and sorafenib (platelet-derived growth factor receptor and vascular endothelial growth factor receptor) showed no change on DEPTOR Tyr phosphorylation (Fig. 6*B*). Finally, we tested inhibitors against the SRC Tyr kinase family (PP2, dasatinib, SRC kinase inhibitor I, and SU6656) and observed a significant decrease of DEPTOR Tyr phosphorylation following PP2 treatment but did not detect any significant response with other inhibitors of this family (Fig. 6*C*). The PP2-mediated decrease in DEPTOR Tyr phosphorylation was more noticeable at concentrations over 1 μM (Fig. S3*B*). Similarly, to results obtained using PP242, the requirement of higher doses of PP2 suggested that the effect was most probably not because of inhibition of SRC kinase family members but from other kinases potentially sensitive to PP2-mediated inhibition at higher concentration (Table S2) (36, 37, 38, 39, 40, 41).

### EPH receptors induce DEPTOR Tyr 289 phosphorylation

**Figure 7:**
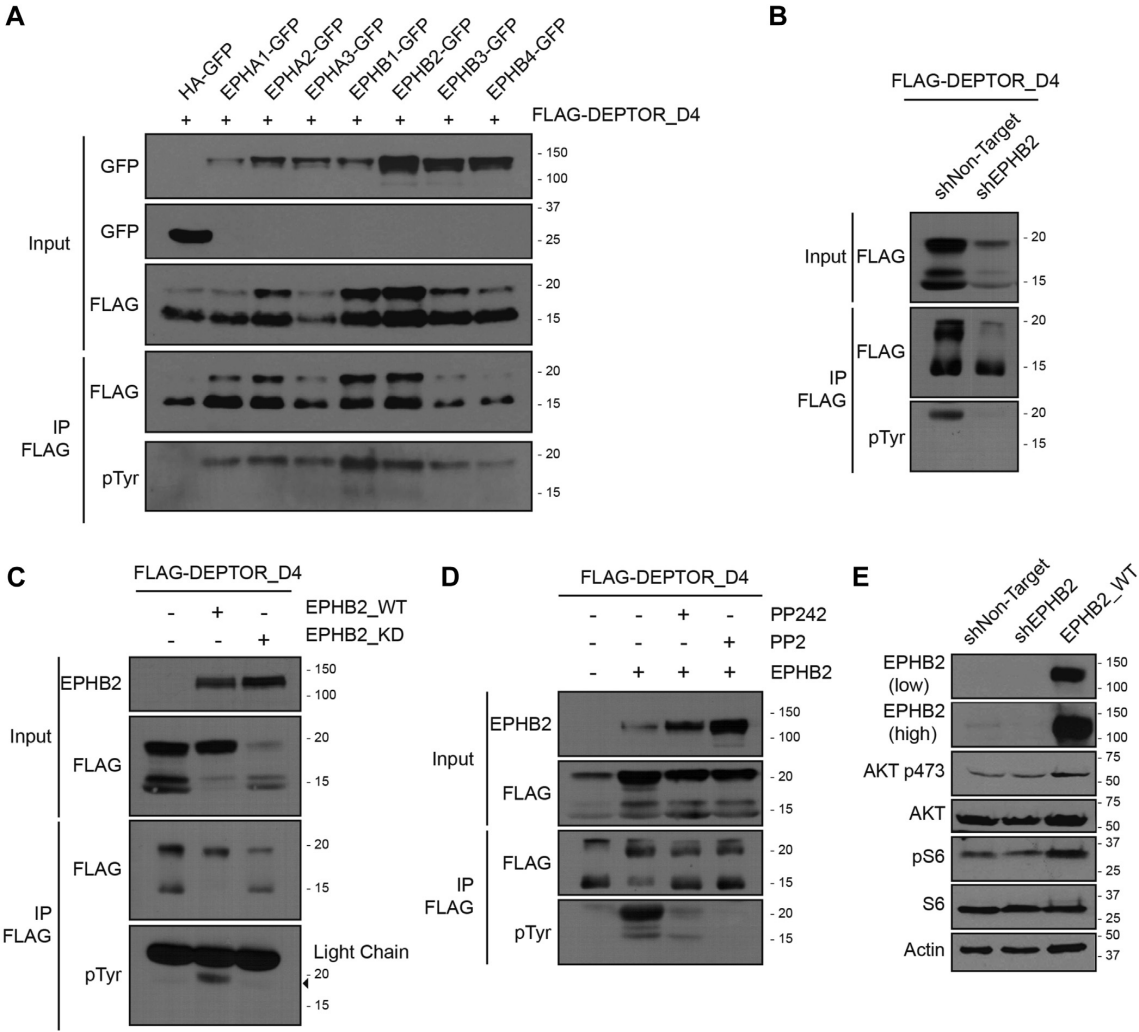
**EPHB2 promotes DEPTOR tyrosine 289 phosphorylation and mTOR activation.***A*, 293T cells were transfected with FLAG-DEPTOR-D4 in combination with HA-GFP, EPHA1-GFP, EPHA2-GFP, EPHA3-GFP, EPHB1-GFP, EPHB2-GFP, EPHB3-GFP, or EPHB4-GFP. Cells were lysed for a denaturing FLAG-tag immunoprecipitation to reveal the levels of tyrosine phosphorylation of DEPTOR. *B*, 293T cells and EPHB2-depleted 293T cells were transfected with FLAG-DEPTOR-D4 and lysed to perform a FLAG-denaturing immunoprecipitation to reveal the levels of DEPTOR-D4 tyrosine phosphorylation. *C*, EPHB2-depleted 293T cells were transfected with FLAG-DEPTOR-D4 in combination with EPHB2^WT^ or EPHB2^KD^ (kinase dead). Cells were lysed to perform a FLAG-denaturing immunoprecipitation to reveal the levels of DEPTOR-D4 tyrosine phosphorylation. *D*, 293T cells transfected with FLAG-DEPTOR-D4 in combination with EPHB2^WT^ were treated with PP242 (10 μM) or PP2 (10 μM) for 2 h before cells lysis. FLAG-denaturing immunoprecipitation to reveal the levels of DEPTOR-D4 tyrosine phosphorylation. *E*, lysate from EPHB2-depleted or EPHB2^WT^-overexpressing HeLa cells were used to assess mTOR activity. HA, hemagglutinin; mTOR, mechanistic target of rapamycin.

Of the possible secondary targets of PP242 and PP2 inhibition, only EPH Tyr kinase receptors were previously reported to be inhibited at high concentrations of the two drugs (37, 42, 43). To identify which EPH receptor is responsible for DEPTOR Tyr phosphorylation, we coexpressed different EPH receptors in 293T cells. We found that both EPHB1 and EPHB2 significantly increased the level of DEPTOR-D4 Tyr phosphorylation (Fig. 7*A*). Conversely, EPHB2 depletion (Fig. S4*A*) led to a decrease in Tyr phosphorylation (Fig. 7*B*). In addition, we validated that EPHB2 overexpression increased Tyr phosphorylation on full-length DEPTOR (Fig. S4*B*), and that Tyr 289 was the residue targeted by EPHB2 (Fig. S4*C*). As EPHB2 seems to be a major regulator of DEPTOR Tyr phosphorylation, we observed that a kinase-dead EPHB2 mutant (K653A) failed to phosphorylate DEPTOR when overexpressed (Fig. 7*C*). Moreover, depletion of EPHB2 or overexpression of the kinase-dead version decreased the cellular levels of DEPTOR (Fig. 7, *B* and *C*). Hence, we proceeded to confirm that preventing DEPTOR Tyr phosphorylation, through impairing the EPHB2 pathway, decreased its protein stability. As expected, we observed a significant decrease in DEPTOR half-life in the presence of kinase dead EPHB2, when compared with WT, following growth factor stimulation (Fig. S4*D*).

Using the EPHB2 overexpressing or depleted cells, we assessed PP242 and PP2 ability to repress EPH receptor–induced DEPTOR Tyr phosphorylation. In EPHB2-overexpressing cells, both inhibitors drastically decreased DEPTOR (Fig. 7*D*) and EPHB2 (Fig. S4*E*) Tyr phosphorylation, while we observed a synergistic reduction of the phosphorylation in cells expressing EPHB2-targeting shRNAs (Fig. S5, *A* and *B*). These results suggest that other Tyr kinase(s) can at least partially compensate for the loss of EPHB2 involved in DEPTOR Tyr phosphorylation (Figs. 7*D* and S5, *B* and *C*). To assess the effect of EPHB2 on the activation levels of both mTOR complexes, we either overexpressed or depleted EPHB2^WT^ in HeLa cells (Fig. 7*E*). EPHB2^WT^-overexpressing cells showed increased mTORC1 and mTORC2 activation when compared with both shNT and shEPHB2 cells, confirming that EPHB2 levels can affect mTOR activation thorough DEPTOR phosphorylation.

### Identification of SYK as a potential kinase for DEPTOR Tyr phosphorylation

**Figure 8:**
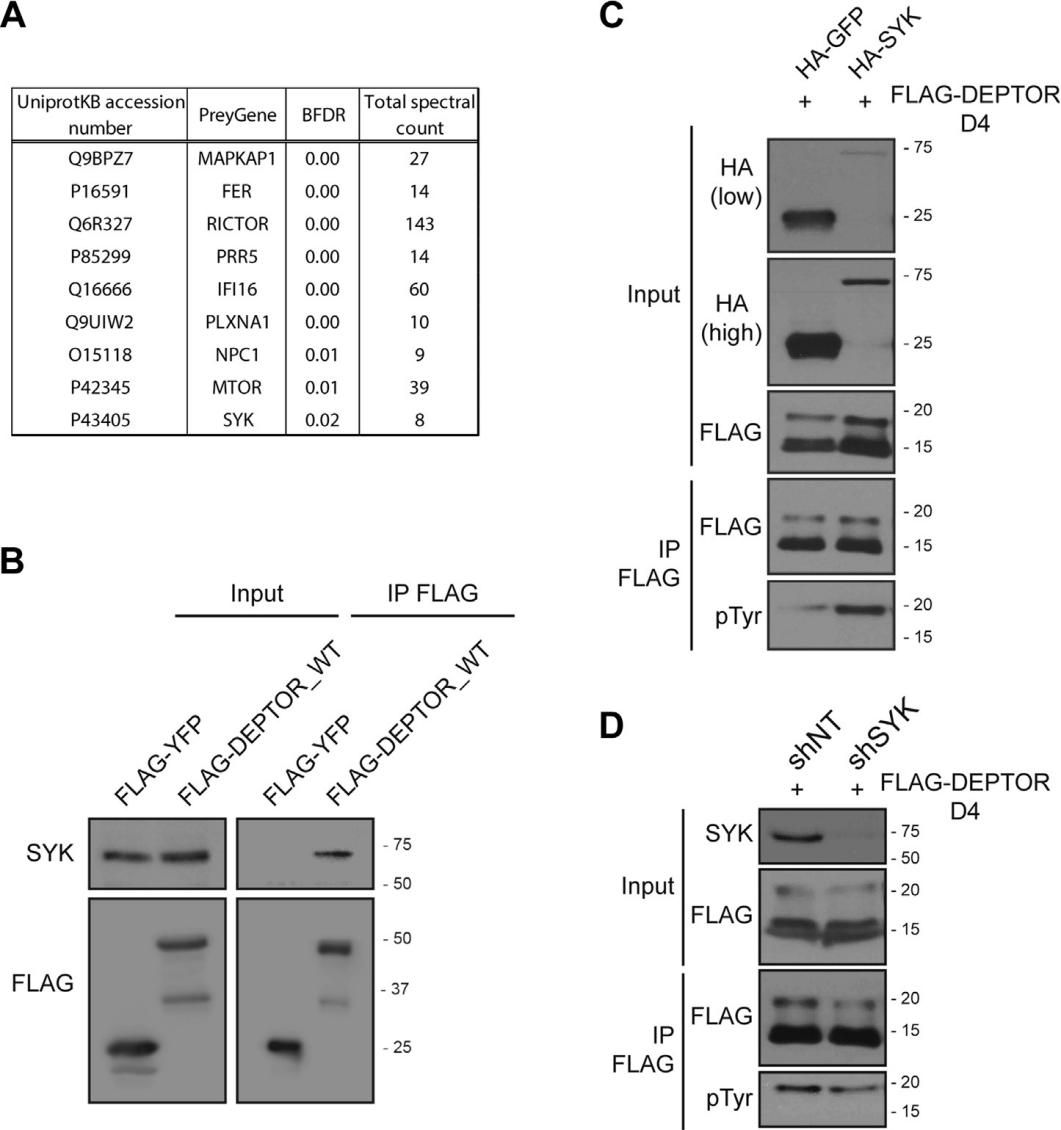
**SYK associates with DEPTOR and promotes its tyrosine phosphorylation.***A*, BFDR MS analysis of the DEPTOR_D4 fragment MiniTurbo assays. *B*, SYK detection in FLAG immunoprecipitate made using 293T cells overexpressing FLAG-YFP and FLAG-DEPTOR_D4^WT^. *C*, 293T cells overexpressing FLAG-DEPTOR_D4 and a combination of either HA-GFP or HA-SYK were lysed for a denaturing FLAG immunoprecipitation to reveal DEPTOR_D4 tyrosine phosphorylation levels. *D*, 293T cells expressing a Ctrl shRNA of a SYK shRNA were transfected with FLAG-DEPTOR_D4. Cells were lysed for a denaturing FLAG immunoprecipitation to reveal DEPTOR_D4 tyrosine phosphorylation levels. BFDR, Bayesian false discovery rate; HA, hemagglutinin; SYK, spleen tyrosine kinase.

As we were unable to confirm direct interaction between EPHB2 and DEPTOR (data not shown), we reasoned that DEPTOR Tyr phosphorylation was mediated through another Tyr kinase activated by EPHB2 receptor. To identify the potential kinase able to phosphorylate the Tyr 289 of DEPTOR, we performed a miniturboID experiment using DEPTOR D4 fragment as bait. We first validated our constructs by evaluating their capacity to biotinylate partners (Fig. S6*A*) and by revealing their autobiotinylation following streptavidin pull down (Fig. S6*B*). We then proceeded with duplicate biotinylated assays and identified, among all the potential interactors (Fig. 8*A*), two Tyr kinases: Fer (Feline sarcoma–related protein, Fer) and SYK. Since SYK was already shown to interact with EPH receptors (44), as well as the mTOR pathways (45), we first confirmed the interaction between SYK and DEPTOR (Fig. 8*B*). We then assessed if SYK could phosphorylate DEPTOR Tyr 289 by either overexpressing or abrogating SYK expression, and then assessed the level of phosphorylation of DEPTOR. As expected, SYK overexpression greatly increased the level of Tyr phosphorylation on DEPTOR (Fig. 8*C*), whereas decreasing SYK expression, using a validated shRNA (Fig. S7*A*), showed the opposite effect (Fig. 8*D*). We further confirmed these observations using DEPTOR full length (Fig. S7*B*) and the specificity of the phosphorylation site by using DEPTOR D4^Y289F^ fragment (Fig. S7*C*).

**Figure 9:**
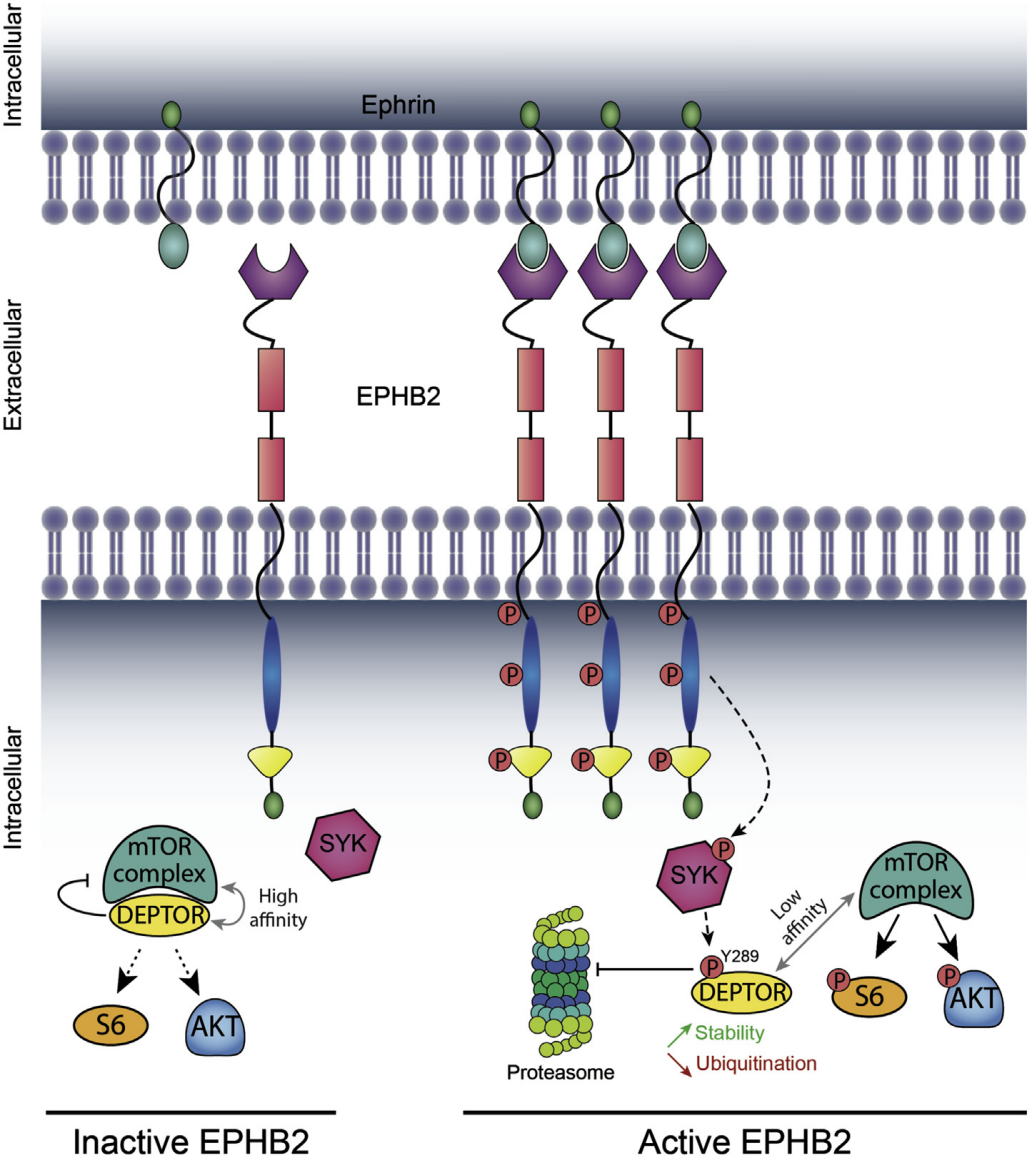
**Model of mTOR activation by EPHB2-regulated DEPTOR tyrosine 289 phosphorylation.** Binding of ephrins to EPHB2 receptors causes their clustering and activation. Active EPHB2 receptors phosphorylate the tyrosine kinase SYK, causing its activation and the phosphorylation of DEPTOR on its tyrosine 289. This latter phosphorylation event increases DEPTOR stability, whereas decreasing its affinity to mTOR, leading to an increased and sustained activation of mTOR. Conversely, inactivated EPHB2 receptors limit DEPTOR tyrosine 289 phosphorylation, which allows DEPTOR binding to mTOR, thus inhibiting its activity. mTOR, mechanistic target of rapamycin; SYK, spleen tyrosine kinase.

Taken together, our results exposed a new mTORC1 and mTORC2 regulatory mechanism mediated by the activation of EPHB2 receptors and SYK, which promote the phosphorylation of DEPTOR. This phosphorylation of DEPTOR promotes its dissociation from mTOR complexes, thus allowing the rapid and sustained activation of mTORC1/mTORC2 (Fig. 9).

## Discussion

mTOR is frequently hyperactivated in cancer, conferring cancer cells with proliferative and survival advantages over normal cells (13). While this scenario is usually associated with mutations within the PTEN/PI3K/AKT regulatory pathways, some cancers were shown to progress through dysregulation of DEPTOR inhibitory functions (23). Most of these cases reported an increased DEPTOR degradation rate through the proteasomal degradation pathway, resulting in decreased total cellular DEPTOR levels (24, 25, 26). While DEPTOR can be defined as a fine tuner of mTOR functions, its downregulation is enough to promote unchecked mTORC1 and mTORC2 activation in a PTEN/PI3K/AKT-independent manner (17, 18).

Herein, we propose a novel DEPTOR regulatory mechanism that does not involve its proteasomal degradation. Indeed, we found that DEPTOR Tyr^289^ phosphorylation can decrease its affinity toward both mTOR complexes (mTORC1 and mTORC2), which lead to increased mTOR activation, while increasing DEPTOR stability. This represents a novel mode of DEPTOR regulation contrasting with the reported mechanism where DEPTOR dissociation to mTOR was initiated by serine phosphorylation, which subsequently leads to its proteasomal degradation (24, 25, 26). These elements suggest that different phosphorylation events can distinctly modulate the DEPTOR regulatory function, but that phosphorylation on Tyr 289 might act as a rapid and reversible inhibitory mechanism preventing its proteasome-mediated degradation. As Tyr 289 lies within an amino acid sequence containing key serine residues (Ser 286/287/291/293/299) shown to regulate DEPTOR degradation (24, 25, 26), it is possible that Tyr 289 phosphorylation can interfere with CK1α/β-TRCP–mediated degradation of DEPTOR. While further experiments are needed to confirm this hypothesis, many studies have shown a complex interplay between PTM (46), suggesting that DEPTOR Tyr 289 phosphorylation of DEPTOR can potentially block other PTMs, such as methylation, ubiquitination, and Ser/Thr phosphorylation. This relationship with other PTMs is intriguing, and DEPTOR C-terminal and DEPTOR D4 fragment seems to be more permissive for them, probably caused by the accessibility of the site comparatively to DEPTOR full length. Future work will be necessary to assess the impact of PTM on each other.

Based on our observations, Tyr 289 phosphorylation not only stimulated DEPTOR dissociation from the mTOR complexes but also interfered with its reassociation within mTOR complexes. Indeed, the phosphomimetic Y289E DEPTOR mutant was found to maintain increased mTOR activation through time, whereas the unphosphorylatable Y289F mutant was more potent to inhibit both complexes when compared with DEPTOR^WT^. This mechanism of activation could act as a molecular switch allowing the rapid activation or repression of mTOR, since a Tyr phosphorylated DEPTOR might reversibly be dephosphorylated to inhibit mTOR. The rapid alternance between an active and inactive form of mTOR can thus be achieved more efficiently than the already observed cycle of degradation/translation of DEPTOR caused by CK1α/β-TRCP signaling pathways.

Recent advances on DEPTOR regulation mechanisms have led to a model of retroactive feedback effect of DEPTOR on mTOR where DEPTOR can directly inhibit mTORC1, whereas its effect on mTORC2 was indirectly caused by PI3K activation (47). However, our results show that DEPTOR Tyr 289 phosphorylation affects the dynamics for mTORC2 activation/inhibition more rapidly than mTORC1, suggesting a dual mode of regulation of mTORC2 activity by DEPTOR. Indeed, some studies seem to contradict this PI3K-mediated mTORC2 inhibitory feedback model (48, 49). While it was suggested that this is dependent on cellular context (*i.e.*, mutation of the PI3K/PTEN pathway), it also seems possible that mTORC2 activation was regulated by the DEPTOR Tyr 289 phosphorylation. This is further supported by our observation that DEPTOR Tyr 289-mediated regulation of mTOR bypasses the PI3K/PTEN pathway. While this remains to be validated, DEPTOR Tyr 289 phosphorylation could have been oversighted in their assessment of mTORC2 activation, thus corroborating the existence of two modes of regulation by DEPTOR, one going through the retroactivation of PI3K and a transient one going through the phosphorylation of DEPTOR Tyr 289.

While studies already revealed a possible link between EPH receptors and mTOR pathways, we observed that the effect of EPH receptors on mTOR activation or inhibition was highly specific. Some previous studies indicated that EPHA receptor can inhibit the mTOR pathway (50, 51), whereas others indicated that EPHB2 can activate it (39, 52). Hence, the differential cellular response described previously could be dictated by expression levels of specific EPH receptors in the cell lines used. In our current study, we focused on EPHB2, as it was shown to be involved in cellular processes known to be driven by mTORC1 and mTORC2 activation (38) and that EPHB2 was shown to increase the phosphorylation of PI3K and AKT, resulting in mTOR activation (39, 52). Our results corroborate these observations, as EPHB2 expression increased DEPTOR Tyr 289 phosphorylation, whereas its depletion decreased it. Future work should include a larger spectrum of cell lines to assess mTOR activation levels in cells where EPHB2 is abundant.

Previous reports linked SYK to mTOR activation by the PI3K/AKT pathway (45). However, our work showed a direct link by the promotion of the phosphorylation of the endogenous inhibitor of mTOR (*e.g.*, DEPTOR), explaining how SYK mediates mTOR activation independently of the PI3K/AKT axis (53). Based on the diversity and complexity of mTOR functions, it is not surprising that alternative regulatory mechanisms remain unknown.

Mutations on DEPTOR Tyr 289 have not been reported in cancer, whereas mutations within EPHB2 and SYK in various malignancies were described in multiple publications (38, 54, 55, 56, 57). Thus, it remains possible that the regulation of DEPTOR on mTOR can be made through Tyr 289 phosphorylation in tumors harboring altered EPHB2 or SYK. Moreover, while the link between EPH and SYK remains to be further defined, our results uncovered a novel regulatory mechanism allowing the rapid modulation of mTOR activity, through the phosphorylation of a single Tyr within the DEPTOR C terminus (Tyr 289). This EPH receptor/SYK/DEPTOR mechanism, which bypasses the canonical pathway of mTOR activation, could potentially explain why so many cancers show constitutive mTOR activation without mutations in the PI3K/AKT pathway.

## Experimental procedures

### Cell culture

293T, HeLa, and normal human astrocytes immortalized by hTERT and 293E were cultured in Dulbecco's modified Eagle's medium (Sigma) supplemented with 10% fetal bovine serum (Gibco) and 1% penicillin/streptomycin (Wisent).

### Plasmids and transfections

Cells were transfected with 8 μg of DNA using 30 μg of linear polyethylenimine. The following vectors were used: pCDNA3.1 FLAG-eYFP, pRK5 FLAG-DEPTOR, pLJM1 FLAG-DEPTOR-D1/D2, pLJM1 FLAG-DEPTOR-D3/D4, pLJM1 FLAG-DEPTOR-D4 (WT, Y289F, Y289E, Y326F, Y289/326F), pLJM1 FLAG-DEPTOR-D4-HA (WT, Y289F, Y289E), pLJM1 HA-GFP, pLJM1 EPHA1-GFP, pLJM1 EPHA2-GFP, pLJM1 EPHA3-GFP, pLJM1 EPHB1-GFP, pLJM1 EPHB2-GFP, pLJM1 EPHB3-GFP, pLJM1 EPHB4-GFP, pCDNA5 EPHB2 Wt, pCDNA EPHB2 KD, and pLJM1 HA-SYK.

### Lentiviral transductions

Lentiviral transductions were performed similarly as previously described (58, 59, 60). Briefly, lentiviral particles were generated by transfecting 293T cells using 12 μg of shRNA plasmid (pLKO.1 puro was a gift from Bob Weinberg; Addgene; plasmid no. 8453; http://n2t.net/addgene:8453; RRID: Addgene_8453) (61) or expression plasmid pLJM1 (pLJM1-EGFP was a gift from David Sabatini [Addgene; plasmid no. 19319; http://n2t.net/addgene:19319; RRID: Addgene_19319]) (62), 6 μg of psPAX2 packaging plasmid (psPAX2 was a gift from Didier Trono [Addgene; plasmid no. 12260; http://n2t.net/addgene:12260; RRID: Addgene_12260]), and 2 μg pMD2.G envelope plasmid (pMD2.G was a gift from Didier Trono (Addgene; plasmid no. 12259; http://n2t.net/addgene:12259; RRID: Addgene_12259). Media were changed 24 h after transfection, and lentiviral particles were harvested 24 h later. Viral supernatant was filtered through 0.45 μm filters and supplemented with 8 μg/ml polybrene and 10% fetal bovine serum. The following plasmids were used: pLKO.1-sh_NT, pLKO.1-shGFP, pLJM1 FLAG-DEPTOR (WT, Y289F, Y289E), pLJM1 FLAG-GFP, pLKO shDEPTOR_3, pLKO shDEPTOR_5, pLKO shEPHB2_GFP_3, pLKO shEPHB2_GFP_5, pLKO shSYK_1, pLKO shSYK_2, pLJM1_FLAG_GFP_miniturbo, pLJM1_FLAG-DEPTOR_D4-miniturbo.

### RNA interference

DEPTOR shRNA_3 (5′-CCGGTAGAAAGGTGTTGAAATGCTTCTCTATCAAGAGTAGAGAAGCATTTCAACACCTTTCTTTTTTTG-3′), DEPTOR shRNA_5 (5′-CCGGTGCAGCTATTTAATAGGGAATCTAGATCAAGAGTCTAGATTCCCTATTAAATAGCTGCTTTTTTG-3′), EPHB2 shRNA_3 (5′-CCGGTTTAAAGAGGATTCTCATAAGCTCGAGCTTATGAGAATCCTCTTTAAATTTTTTG-3′), EPHB2 shRNA_5 (5′-CCGGGCTAGACAAGATGATCCGCAACTCGAGTTGCGGATCATCTTGTCTAGCTTTTT-3′), SYK shRNA_1 (5′-CCGGTCAGAGGAATTTGGCTGCTTCTTCAAGAGAGAAGCAGCCAAATTCCTCTGTTTTTTG-3′), SYK shRNA_2 (5′-CCGGTGTGATTGCACTTGGACATCAGTCCATCAAGAGTGGACTGATGTCCAAGTGCAATCACTTTTTTG-3′), and shRNA nontarget (5′-CCGGGCGCGATAGCGCTAATAATTTCTCGAGAAATTATTAGCGCTATCGCGCTTTTTG-3′).

### Cell lysis, immunoblots, and antibodies

Cells were lysed in Laemmli buffer, proteins were separated on SDS-PAGE, and transferred to polyvinylidene fluoride membranes (Bio-Rad). Primary antibodies used were anti-FLAG (2368; NEB; 1:1000 dilution), anti-HA (3724; NEB; 1:1000 dilution), anti-mTOR (7C10; Cell Signaling; 1:1000 dilution), anti-DEPTOR (D9F5; Cell Signaling; 1:1000 dilution), anti-S6 (54D2; Cell Signaling; 1:1000 dilution), anti-phospho-S6^S240/244^ (2215; Cell Signaling; 1:1000 dilution), anti-pan-AKT (C67E7; Cell Signaling; 1:1000), antiphospho-AKT^S473^ (D9E; Cell Signaling; 1:1000 dilution), anti-ACTIN (D6A8; Cell Signaling; 1:2000 dilution) and anti-GAPDH (D16H11; Cell Signaling; 1:2000 dilution), anti-4G10 (05-321; Millipore–Sigma; 1:1000 dilution), anti-GFP (2956; Cell Signaling; 1:1000 dilution), antiubiquitin P4D1 (sc-8017; Santa Cruz; 1:1000 dilution), anti-EPHB2 D2X2I (83029; Cell Signaling; 1:1000 dilution), anti-EPHB2 (AF467; R&D Systems; 1:200 dilution), and anti-SYK D3ZE1 (13198; Cell Signaling; 1:500 dilution).

### IPs

For nondenaturing IP, after specific treatment, cells were lysed in 1 ml of lysis buffer (150 mM NaCl, 1% Nonidet P-40, and 25 mM Tris [pH 7.4]) and incubated on ice for 10 min. After vortex, lysate was centrifuged for 10 min at 17,000*g* at 4 °C. The supernatant was incubated for 1 h at 4 °C with 15 μl of FLAG M2 agarose beads (Sigma). Immunoprecipitates were washed three times with lysis buffer containing before elution in Laemmli buffer. For denaturing IP, after specific treatment, cells were lysed in 1 ml of denaturing buffer (1% SDS, 5 mM EDTA, and 10 mM beta-mercaptoethanol), boiled for 5 min, and sonicated with FISHER SONIC dismembrator model 150 at level 6 for 20 s. All lysates were complemented with 9 ml of nondenaturing buffer (20 mM Tris–HCl [pH 8], 137 mM NaCl, 1% Triton X-100, and 2 mM EDTA) and 15 μl of FLAG M2 agarose beads (Sigma) and incubated for 1 h at 4 °C. Immunoprecipitates were washed three times with nondenaturing buffer before elution in Laemmli buffer.

### Phosphatase treatment

After washes of denaturing IP, beads were washed twice in PBS and separated in two. One part was treated 30 min at 37 °C with only calf intestinal alkaline phosphatase (CIP) buffer (NEB CutSmart buffer; 1×), and the other one was treated with CIP buffer and CIP phosphatase (M0525; NEB).

### Microscopy mean cell area determination

Cells were plated on Ibidi plates (81156; Ibidi) and starved for 16 h. Stimulation treatments with complete media were done, and cells were fixed with 4% formaldehyde at different time points (starved, 15 min, 3, 6, 12, and 18 h stimulation) after three washes of PBS 1×. After fixation, cells were washed five times with PBS 1× and permeabilized with PBS 0.5% Triton for 20 min. Five washes were made, and cells were incubated at room temperature for 1 h with PBS–Tween bovine serum albumin 0.2%. Cells were washed five times with PBS–Tween and incubated for 1 h at room temperature with an anti-FLAG M2 (F3155; Sigma; 1:20,000) diluted in PBS 1×. After five washes with PBS–Tween, cells were incubated 1 h with an anti-Alexa-Fluor 488 (4409; NEB; 1:200 dilution), phalloidin (8953S; VWR; 1:400 dilution), and 4′,6-diamidino-2-phenylindole (D9524-10MG; Sigma–Aldrich; 1:500 dilution). About ten washes were made (five washes with PBS–Tween and five washes with PBS 1×) and kept in PBS 1× until imaging. Total internal reflection fluorescence microscope was used to image the experiment. Large images were taken (8 × 8) with a 15% stitching between each image at 20×. Images were then analyzed using NIS Element Analysis software. The mean area of the cells was calculated for each condition (n = 3, biological replicates).

### Measurement of mTOR activity

Cells were starved for 16 h and stimulated 30 min with complete media. After the stimulation, they were starved again during 1, 2, 3, 4, 6, 8, and 12 h or during 5, 15, 30, 45, 60, 120, and 180 min. Cell extracts were also made for starve and 30 min stimulation condition.

### MiniTurboID experiment

Infected 293T cells with FLAG-GFP-MiniTurbo and FLAG-DEPTOR_D4-MiniTurbo were treated 1 h with 50 μM of biotin. Cell lysates were cleared of free biotin using a 3K Microsep advance centrifugal device (MCP003C41; Pall), and the supernatant was used for a denaturing streptavidin pull down. To prepare for MS analysis, the pulled-down biotin-labeled proteins were washed three times with 50 mM of ammonium bicarbonate, resuspended in 100 μl of ammonium bicarbonate with 1 μg of trypsin, and incubated overnight at 37 °C with gentle lateral mixing. About 16 h later, each sample was supplemented with 1 μg of trypsin and incubated for another 4 h. The supernatants were collected, beads were washed twice with 200 μl of MS-grade acetonitrile, and the washes were pooled with the collected supernatant. Digestion was stopped with a concentration of 2% of formic acid. Peptides were desalted with homemade C_18_-StageTips, dried with a SpeedVac, and stored at −80 °C until MS analysis.

### MS data acquisition and analysis

Peptide samples were analyzed at the Sherbrooke mass spectrometry core facility. Briefly, samples were injected into an HPLC (nanoElute; Bruker Daltonics) and loaded onto a trap column with a constant flow of 4 μl/min (Acclaim PepMap100 C_18_ column, 0.3 mm id × 5 mm; Dionex Corporation) and eluted onto an analytical C_18_ column (1.9 μm beads size, 75 μm × 25 cm; PepSep). Peptides were eluted over a 2-h gradient of acetonitrile (5–40%) in 0.1% formic acid at 400 nl/min while being injected into a TimsTOF Pro mass spectrometer equipped with a Captive Spray nano electrospray source (Bruker Daltonics). Data were acquired using data-dependent auto-MS/MS with a 100 to 1700 *m/z* mass range, with parallel accumulation serial fragmentation enabled with a number of parallel accumulation serial fragmentation scans set at 10 (1.27 s duty cycle) and a dynamic exclusion of 0.4 min, *m/z*-dependent isolation window, and collision energy of 42.0 eV. The target intensity was set to 20,000, with an intensity threshold of 2500. The resulting files were analyzed using MaxQuant (software package, version 1.6.17.0) using a mass tolerance of 20 ppm for precursor ions and a tolerance of 50 ppm for fragment ions and searched against the UniProt human reference genome (21/03/2020; UP000005640) containing 75,776 entries. Two miscleavages were allowed, while enzymes were set to trypsin (K/R not before P). Carbamidomethylation of cysteine was set as a fixed modification. Phosphorylation of serine, threonine, and Tyr; oxidation of methionine; and acetylation of N-terminal protein were allowed as variable modifications. Peptide-spectrum matches and protein false discovery rates were both applied at 1%. Please note that values of parameters in MaxQuant have not been changed from their default values unless explicitly stated. The complete protein list identified by MaxQuant can be found in Table S3. SAINTexpress was performed using the REPRINT (https://reprint-apms.org/) portal using default parameters. The results of this analysis can be found in Table S4.

## Data availability

All MS files were deposited at MassIVE (massive.ucsd.edu) and assigned the identified MSV000087055. They can be downloaded at ftp://MSV000087055@massive.ucsd.edu.

## Supporting information

This article contains supporting information.

## Conflict of interest

The authors declare that they have no conflicts of interest with the contents of this article.
