# Supplementary material for: Tyrosine phosphorylation of DEPTOR functions as a molecular switch to activate mTOR signaling
Source: J Biol Chem. 2021 Oct 9;297(5):101291. doi: 10.1016/j.jbc.2021.101291 (PMC8551655; doi:10.1016/j.jbc.2021.101291)
Supplement: Supplemental Figures S1–S7 and Tables S1, S2 [file mmc1.pdf]

SUPPLEMENTAL FIGURE LEGENDS

Tyrosine Phosphorylation of DEPTOR Functions as a Molecular Switch to Activate mTOR Signaling

Figure S1. Tyrosine 289 of DEPTOR is phosphorylated.

A) 293T cells were transfected with FLAG-YFP, FLAG-DEPTOR-D4-HA or HA-GFP to validate the integrity of the D4 fragment.

B) 293T cells transfected with FLAG-YFP (control) and FLAG-DEPTOR-D4 were lysed for a FLAG-tag denaturing immunoprecipitation. FLAG-DEPTOR-D4 immunoprecipitate were separated in two samples, one of which was treated with phosphatase to confirm DEPTOR-D4 tyrosine phosphorylation.

C) DEPTOR sequences, corresponding to the D4 fragments, from different species were aligned to assess conserved tyrosine residues during evolution (*).

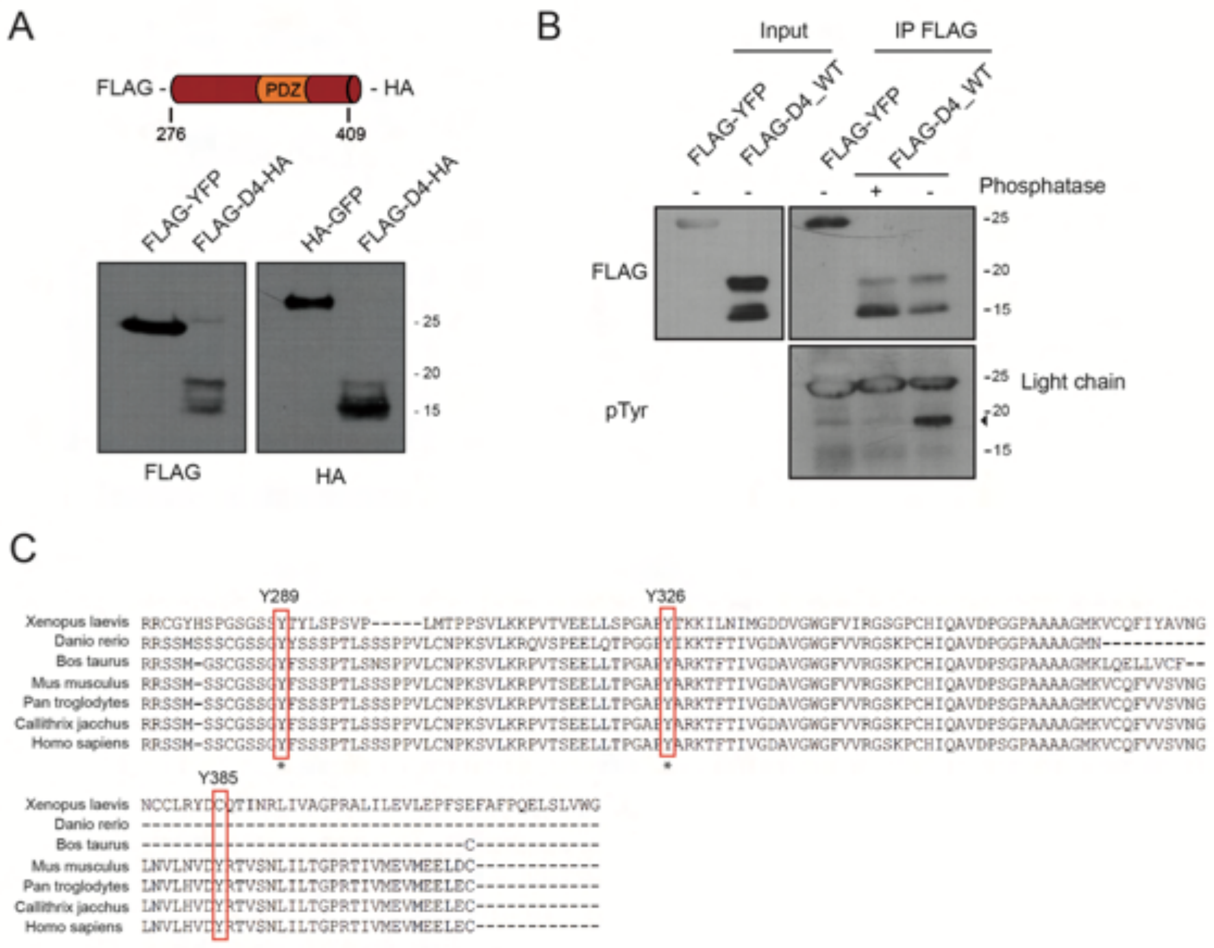

Figure suppl 1

Figure S2. Efficiency of DEPTOR depletion and determining the effect of Tyrosine 289 phosphorylation on β–TRCP1 association.

A) HeLa cells were infected with a Non-target sh (shNT) and two DEPTOR sh (shDEPTOR 3 and 5). DEPTOR protein level was measured in both conditions.

B) b-TRCP1 detection in FLAG immunoprecipitate made using 293T cells overexpressing FLAG-YFP, FLAG-DEPTOR^{WT} and FLAG-DEPTOR^{Y289E}.

A

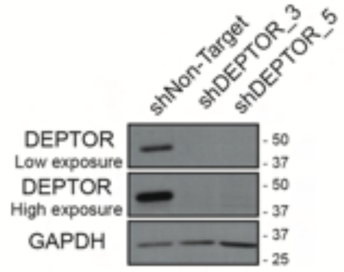

B

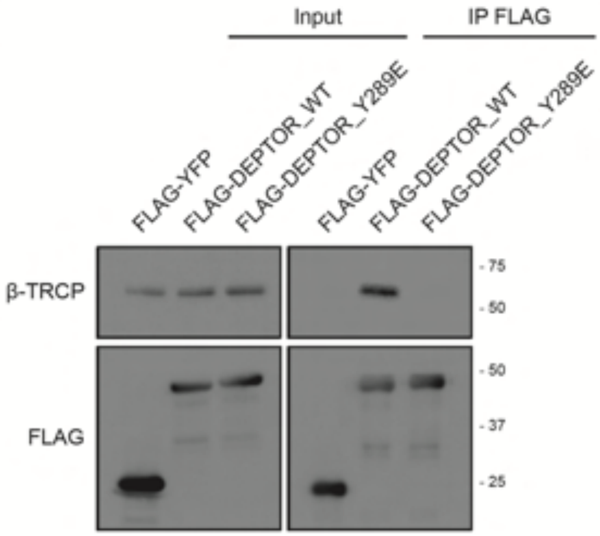

Figure suppl 2

Figure S3. Range of PP242 and PP2 concentration to assess the impact on Y289 phosphorylation.

A) 293T cells were transfected with FLAG-DEPTOR-D4 and treated with DMSO (untreated) or PP242 at increasing concentration (0, 0.01, 0.05, 0.25, 0.5, 1.0, 2.0, 5.0 or 10.0 μM), for 2 hours. Cell lysates were used to perform a denaturing FLAG immunoprecipitation to reveal levels of DEPTOR phosphorylation.

B) 293T cells transfected with FLAG-DEPTOR-D4 were treated with DMSO (untreated) and PP2 at increasing concentration (0, 0.05, 0.5, 0.75, 1.0, 1.5, 2.0, or 10.0 μM), for 2 hours. Cell lysates were used for a denaturing FLAG immunoprecipitation to reveal levels of DEPTOR-D4 phosphorylation.

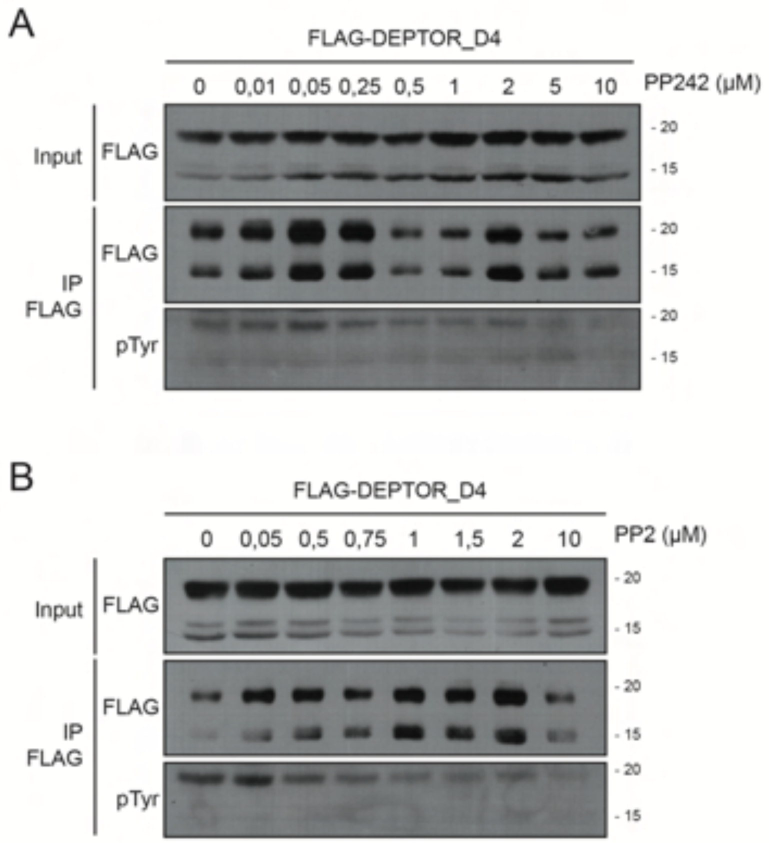

Figure suppl 3

Figure S4. EPHB2 favours DEPTOR tyrosine phosphorylation.

A) CaCo-2 cells were infected with shNT and shEPHB2_3 and shEPHB2_5. Cells were lysed 6 days post-infection to assess EPHB2 expression.

B) 293T cells depleted of EPHB2 were transfected with FLAG-YFP and FLAG-DEPTOR^{WT}, as well as EPHB2. Each lysate was used for a denaturing FLAG immunoprecipitation to assess DEPTOR tyrosine phosphorylation.

C) 293T cells depleted of EPHB2 were transfected with FLAG-YFP, FLAG-DEPTOR-D4^{WT} or FLAG-DEPTOR-D4^{Y289F} in combination with EPHB2. Cells were lysed 48 hours post-transfection and used for a denaturing FLAG immunoprecipitation to assess DEPTOR-D4 tyrosine phosphorylation.

D) EPHB2 depleted HeLa cells were transfected with EPHB2^{WT} or EPHB2^{KD} (kinase dead). Cells were starved for 16 hours, pretreated with 50 µg/ml of cycloheximide for 1 hours, and stimulated with complete media supplemented with cycloheximide (50 µg/ml) for 0, 1, 2, 3, 4 and 6 hours to assess DEPTOR protein levels.

E) 293T cells transfected with EPHB2-GFP were treated with DMSO (untreated), PP242 (10 µM) or PP2 (10 µM) for 2 hours and cell lysates were made to perform a GFP pull-down and assess its phosphorylation state.

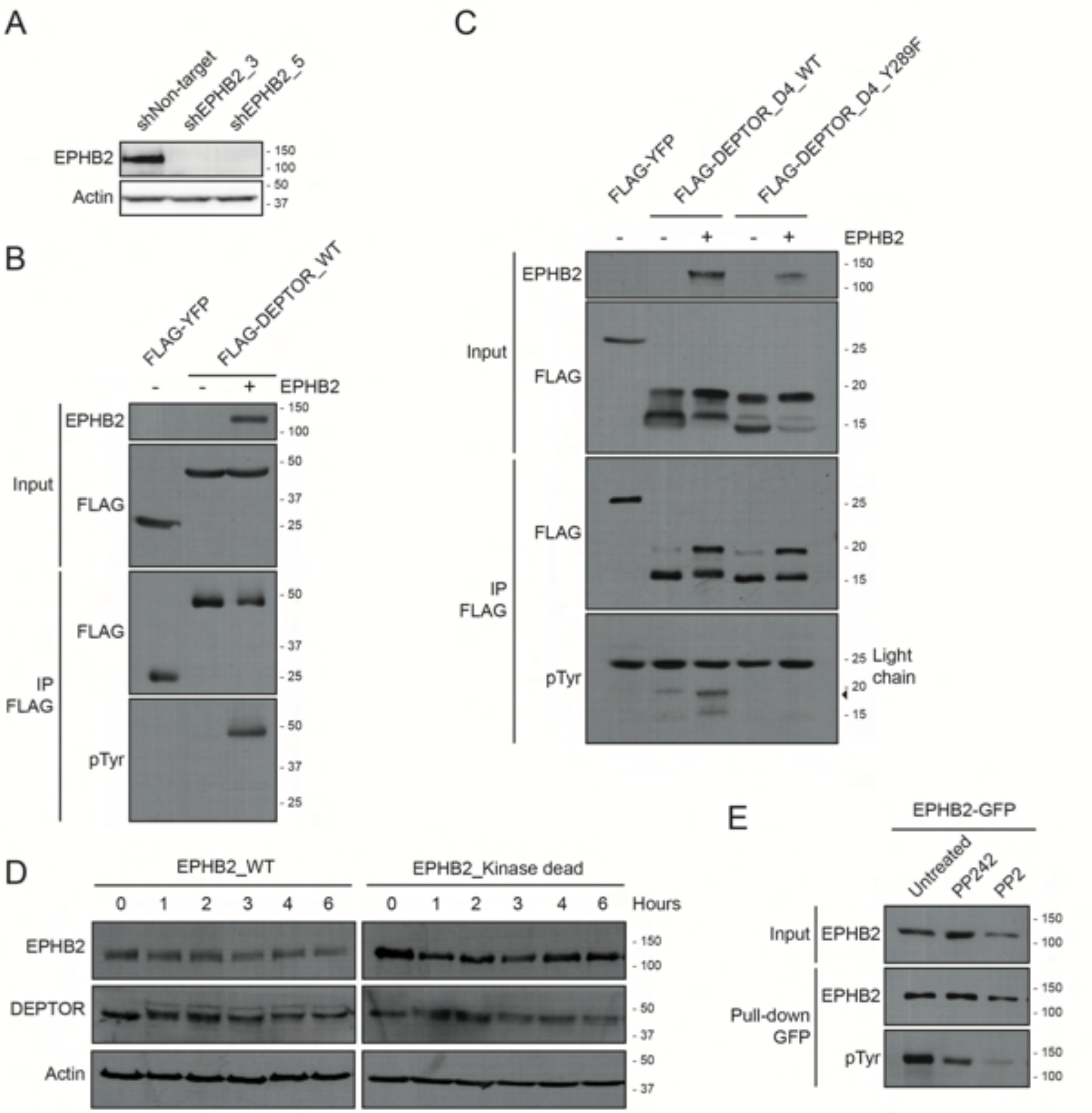

Figure suppl 4

Figure S5. EPHB2 depletion decrease DEPTOR tyrosine phosphorylation.

A) 293T and EPHB2 depleted 293T cells were transfected with FLAG-YFP or FLAG-DEPTOR-D4 and treated with 10 μM of PP242 for 2 hours. Cells were lysed 48 hours post-infection and used for denaturing FLAG-tag immunoprecipitation.

B) 293T and EPHB2 depleted 293T cells were transfected with FLAG-YFP or FLAG-DEPTOR-D4 and treated with 10 μM of PP2 for 2 hours. Cells were lysed and used for denaturing FLAG-tag immunoprecipitation.

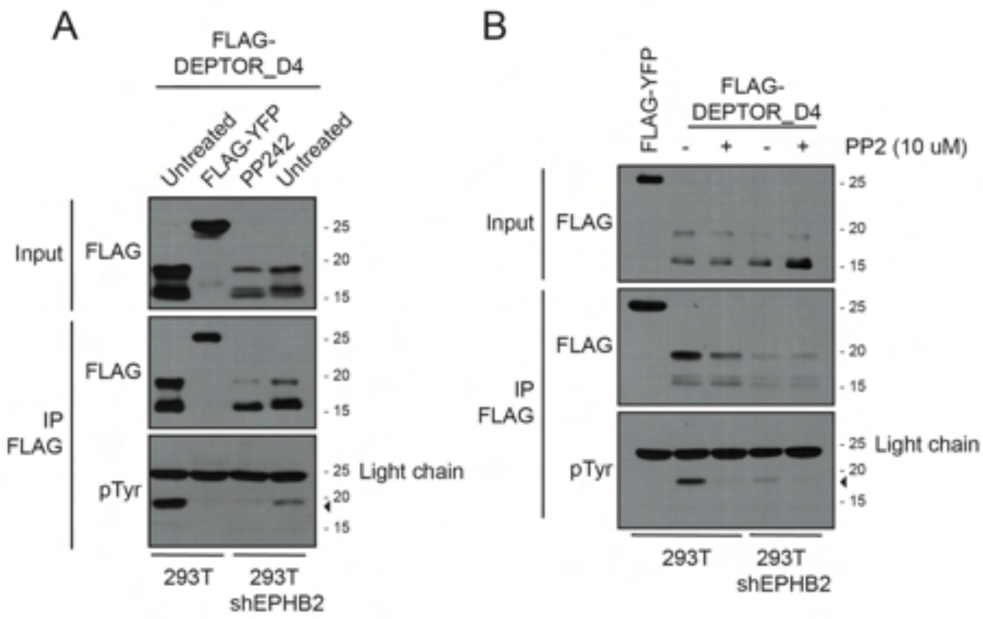

Figure suppl 5

Figure S6. Validation of miniturboID construct.

A) 293T cells infected with FLAG-GFP-MiniTurbo or FLAG-D4-MiniTurbo were incubated or not with 50 μM of biotin for 1 hour. Cells were lysed to reveal biotinylated proteins by streptavidin antibody.

B) 293T cells infected with FLAG-GFP-MiniTurbo or FLAG-D4-MiniTurbo were incubated with 50 μM of biotin for 1 hour. Cells were lysed and used for a streptavidin denaturing immunoprecipitation to assess auto-biotinylation and IP of the differents partners of GFP and D4.

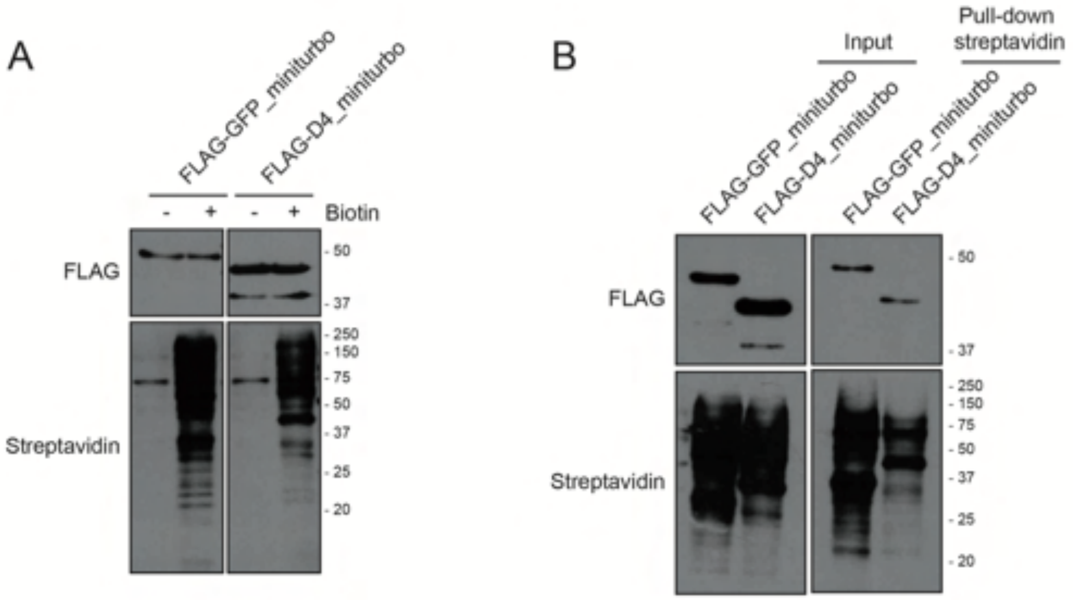

Figure suppl 6

Figure S7. SYK promote tyrosine phosphorylation of DEPTOR

A) 293T cells were infected with shNT, shSYK_1 and shSYK_2. Cells were lysed 48 hours post-infection to assess SYK expression.

B) 293T cells were transfected with FLAG-DEPTOR in combination with HA-GFP or HA-SYK. Cells were lysed 48 hours post-transfection and used for a denaturing FLAG immunoprecipitation to assess DEPTOR tyrosine phosphorylation.

C) 293T cells were transfected with HA-GFP or HA-SYK in combination with FLAG-DEPTOR-D4^{WT} or FLAG-DEPTOR-D4^{Y289F}. Cells were lysed 48 hours post-transfection and used for a denaturing FLAG immunoprecipitation to assess DEPTOR-D4 tyrosine phosphorylation.

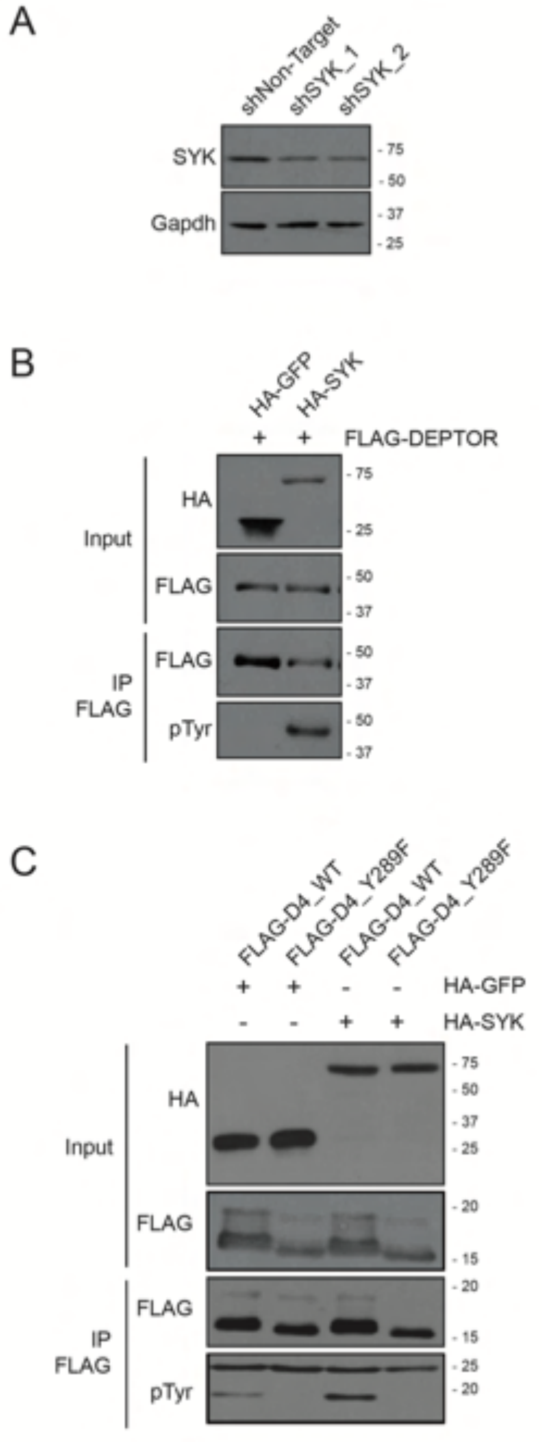

Figure suppl 7

Table S1: PP242 secondary targets and their specific inhibitors.

Table S2: PP2 secondary targets and their specific inhibitors.

	Potential target	IC50	Other inhibitor
PP242 effects	mTOR	30-58 nM	Torin
	PKC	49-198 nM	
	p110	100 nM	Cal-101
	Jak2	110 nM	Ruxolitinib
	Ret	224 nM	Regorafenib
	PDGFR	410 nM	Sorafenib, Imatinib
	DNA-PK	410 nM	Torin, Cal-101
	HcK	1,2 μM	PP2
	Src	1,4 μM	PP2, SU6656, Dasatinib, SKI-I
	VEGFR	1,5 μM	Sorafenib, SKI-I
	EphB4	3,4 μM	NVP-BHG712
	Abl	3,6 μM	Imatinib, Dasatinib
	EGFR	4,4 μM	Lapatinib

Table S1

	Potential target	IC50	Other inhibitor
PP2 effects	Lck	4 nM	SU6656, SKI-I, Dasatinib
	Fyn	5 nM	SU6656, Dasatinib
	Hck	5 nM	PP242
	Rip2	19 nM	SKI-I
	CKIδ	41 nM	D 4476
	Src	100 nM	SU6656, SKI-I, Dasatinib
	EGFR	480 nM	Lapatinib
	CSK	730 nM	SKI-I

Table S2

Table S3: Protein Groups. Protein Groups output from MaxQuant software

Table S4: SAINT express output. SAINT express output from analysis of DEPTOR D4 and GFP miniTurbo analysis

Both Table S3 and Table S4 are available in a single Excel sheet.
